# PharmOmics: A species- and tissue-specific drug signature database and gene-network-based drug repositioning tool

**DOI:** 10.1016/j.isci.2022.104052

**Published:** 2022-03-10

**Authors:** Yen-Wei Chen, Graciel Diamante, Jessica Ding, Thien Xuan Nghiem, Jessica Yang, Sung-Min Ha, Peter Cohn, Douglas Arneson, Montgomery Blencowe, Jennifer Garcia, Nima Zaghari, Paul Patel, Xia Yang

**Affiliations:** 1 Department of Integrative Biology and Physiology, University of California, Los Angeles, Los Angeles, CA 90095, USA; 2 Interdepartmental Program of Molecular Toxicology, University of California, Los Angeles, Los Angeles, CA 90095, USA; 3 Interdepartmental Program of Molecular, Cellular, & Integrative Physiology, Los Angeles, Los Angeles, CA 90095, USA; 4 Interdepartmental Program of Bioinformatics, University of California, Los Angeles, Los Angeles, CA 90095, USA; 5 Institute for Quantitative and Computational Biosciences, University of California, Los Angeles, Los Angeles, CA 90095, USA

**Keywords:** Bioinformatics, Biocomputational method, Systems biology, In silico biology

## Abstract

Drug development has been hampered by a high failure rate in clinical trials due to our incomplete understanding of drug functions across organs and species. Therefore, elucidating species- and tissue-specific drug functions can provide insights into therapeutic efficacy, potential adverse effects, and interspecies differences necessary for effective translational medicine. Here, we present PharmOmics, a drug knowledgebase and analytical tool that is hosted on an interactive web server. Using tissue- and species-specific transcriptome data from human, mouse, and rat curated from different databases, we implemented a gene-network-based approach for drug repositioning. We demonstrate the potential of PharmOmics to retrieve known therapeutic drugs and identify drugs with tissue toxicity using *in silico* performance assessment. We further validated predicted drugs for nonalcoholic fatty liver disease in mice. By combining tissue- and species-specific *in vivo* drug signatures with gene networks, PharmOmics serves as a complementary tool to support drug characterization and network-based medicine.

## Highlights

• Development of PharmOmics, a platform for drug repositioning and toxicity prediction
• Contains >18000 species/tissue-specific gene signatures for 941 drugs and chemicals
• Benchmarked and validated network-based drug repositioning and toxicity prediction
• PharmOmics is freely accessible via an online web server to facilitate user access

## Graphical abstract

**Figure:**
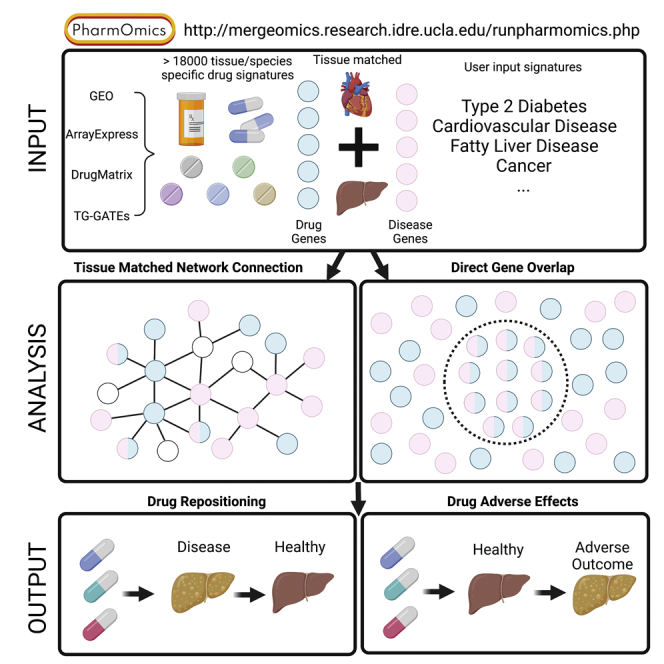

## Introduction

Drug development has been challenging and costly over the past decades due to the high failure rate in clinical trials (Morgan et al., 2011). Most drugs with excellent efficacy and safety profiles in preclinical studies often encounter suboptimal efficacy or safety concerns in humans (Yu, 2016). This translational gap is likely attributable to our incomplete understanding of the systems level activities of drugs in individual tissues and organ systems (Lin and Will, 2012) as well as the differences between humans and preclinical model systems (Denayer et al., 2014).

Drug activities can be captured by gene expression patterns, commonly referred to as gene signatures. By measuring how a pharmacological agent affects the gene signature of a tissue in a particular species, we can infer the tissue-specific biological pathways involved in therapeutic processes or toxicological responses. This concept has prompted drug repositioning studies to repurpose approved drugs for new disease indications (Barabási et al., 2011; Corbett et al., 2012; Hall et al., 2014; Guney et al., 2016; Subramanian et al., 2017; Cheng et al., 2018). Similarly, gene signatures can reveal mechanisms underlying adverse drug reactions (ADRs) and be leveraged to predict ADRs as previously shown for liver and kidney toxicity (Low et al., 2011; Godoy and Bolt, 2012; AbdulHameed et al., 2016).

A drug may affect different molecular processes between tissues, providing treatment effects in the desired target tissue(s) but causing toxicity or ADRs in other tissues, which can be captured in tissue-specific drug signatures. In addition, rodent models have been commonly used in toxicology and preclinical studies, yet species-specific effects of drugs have been observed (Toutain et al., 2010) and may underlie the lack of efficacy or unexpected ADRs of drugs when used in humans (Wong et al., 2018). Therefore, understanding the species-specific molecular effects of drugs is of translational importance. A detailed species- and tissue-specific drug genomic signature database will significantly improve our understanding of the molecular networks affected by drugs at a systems level and facilitate network-based drug discovery and ADR prediction for translational medicine.

The potential of using gene signatures to facilitate target and toxicity identification has led to several major efforts in characterizing genomic signatures related to drug treatment (Wang et al., 2016a, 2016b; Davis et al., 2017; Subramanian et al., 2017). However, none of the existing platforms offer comprehensive cross-tissue and cross-species *in vivo* assessments of drug activities to allow prediction of drug effects on individual tissues and translational potential based on consistencies or discrepancies between species. For instance, the Comparative Toxicogenomics Database (CTD), a literature-based resource curating chemical-to-gene/protein associations as well as chemical-to-disease and gene/protein-to-disease connections (Davis et al., 2017), lacks the cellular and tissue context of the curated interactions. More systematic, data-driven databases such as CMap (Subramanian et al., 2017) and LINC1000 (Wang et al., 2016b) focus on characterizing and cataloging the genomic footprints of more than ten thousand chemicals using *in vitro* cell lines (primarily cancer cell lines) to offer a global view of the molecular responses of individual cellular systems to drugs. However, *in vitro* cell lines may not always capture *in vivo* tissue specificity of drug activities. To move into *in vivo* systems, large toxicogenomics databases such as TG-GATEs (Igarashi et al., 2015) and DrugMatrix from the National Toxicology Program of the National Institute of Environmental Health Sciences (https://ntp.niehs.nih.gov/DrugMatrix/index.html) have become available to provide unbiased transcriptome assessment for heart, muscle, liver, and kidney systems. However, information about other organ systems is limited. Efforts to analyze publicly deposited transcriptomic datasets in GEO (Barrett et al., 2013) and ArrayExpress (Kolesnikov et al., 2015), which have broader tissue coverage, for individual drugs have been described (Wang et al., 2016a), but systematic analyses of species- and tissue-specific effects of drugs have not been achieved.

**Figure 1:**
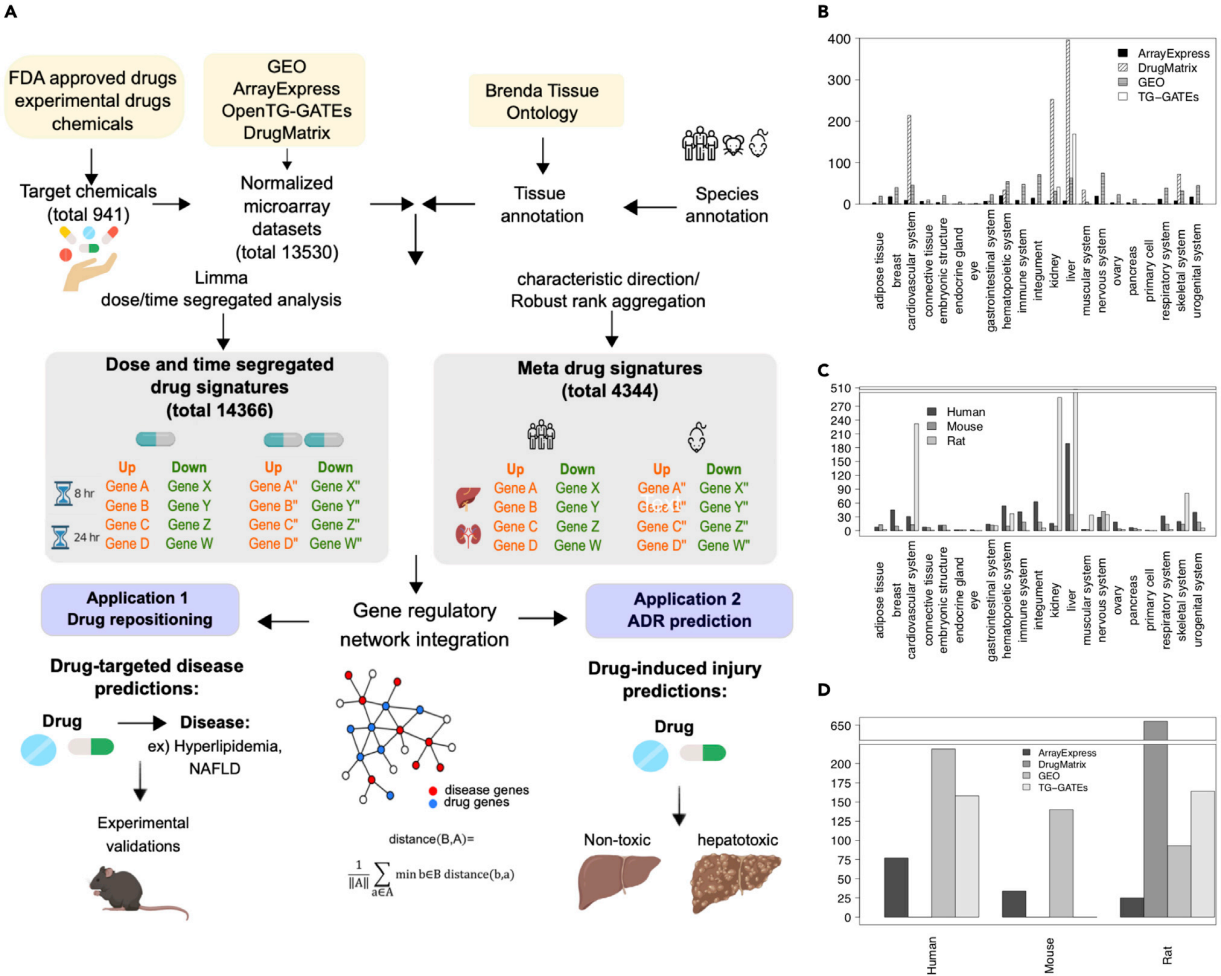
PharmOmics data processing pipeline and database summary (A) FDA-approved drugs were searched against GEO, ArrayExpress, TG-GATEs, and DrugMatrix data repositories. Additional experimental drugs and chemicals from TG-GATEs and DrugMatrix were also included. Datasets were first annotated with tissue and species information, followed by retrieval of dose-/time-segregated signatures using LIMMA (Ritchie et al., 2015) or meta-analysis drug signatures using GeoDE (Clark et al., 2014) and Robust Rank Aggregation (Kolde et al., 2012). These signatures were used to conduct drug repositioning analysis and hepatotoxicity prediction based on either direct gene overlaps or a gene-network-based approach. (B) Summary of available datasets based on data sources and tissues. Y axis indicates unique dataset counts, and X axis indicates tissue and data resources. (C) Summary of available datasets based on tissues and species. Y axis indicates unique dataset counts, and X axis indicates tissue and species. (D) Summary of available datasets based on data sources and species. Y axis indicates unique dataset counts, and X axis indicates data resources and species.

Here, we developed a bioinformatics pipeline (Figure 1) to curate a database that contains 13,530 rat, human, and mouse transcriptomic datasets across >20 tissues covering 941 drugs. We then evaluated the tissue- and species-specificity of drug signatures as well as the performance of these signatures in gene-network-based drug repositioning, toxicity prediction, and comparisons of molecular activities between tissues and species. To benchmark the performance of drug repositioning methods, we conducted *in silico* evaluation of the retrieval rate of known drugs for various diseases, tested method robustness using simulated disease signatures with noise, compared across existing and new methods, and conducted experimental validation of novel predictions. The drug signatures and network-based drug repositioning tool are hosted on an interactive web server, PharmOmics, to enable public access to drug signatures, integrative analyses, and visualization for drug repositioning (http://mergeomics.research.idre.ucla.edu/runpharmomics.php).

## Results

### Construction of the PharmOmics database containing dose-, tissue-, and species-stratified drug signatures

As illustrated in Figure 1A, we compiled a list of clinically relevant drugs, including 766 approved drugs from Kyoto Encyclopedia of Genes and Genomes (KEGG) (Kanehisa et al., 2017), the US Food and Drug Administration (FDA), European Medical Agency, and Japanese Pharmaceuticals and Medical Devices Agency, with an additional 175 chemicals from TG-GATEs (Igarashi et al., 2015) and DrugMatrix (https://ntp.niehs.nih.gov/DrugMatrix/index.html). The compiled drug list was queried against GEO, ArrayExpress, TG-GATEs, and DrugMatrix to identify transcriptomics datasets from human, mouse, and rat studies, which were further annotated with species, tissue, dosage, and treatment time information (STAR Methods). Numbers of datasets, platform information, and sample size distribution are detailed in STAR Methods. Differentially expressed genes (DEGs) were obtained from individual datasets as “dose/time-segregated signatures” and from meta-analysis of multiple datasets for each drug or each class of drugs across treatment regimen for each tissue and each species as “meta-signatures” (STAR Methods). All DEGs are compiled into a drug signature database, comprised of 18,710 gene signatures. Inspection of the database indicated higher coverage for liver compared with other organs/tissues (Figures 1B and 1C), more rat signature sets compared with other species (Figures 1C and 1D), and more signatures from DrugMatrix compared with other data sources (Figures 1B and 1D).

### Implementation of the PharmOmics web server for drug signature query and drug repositioning prediction

**Figure 2:**
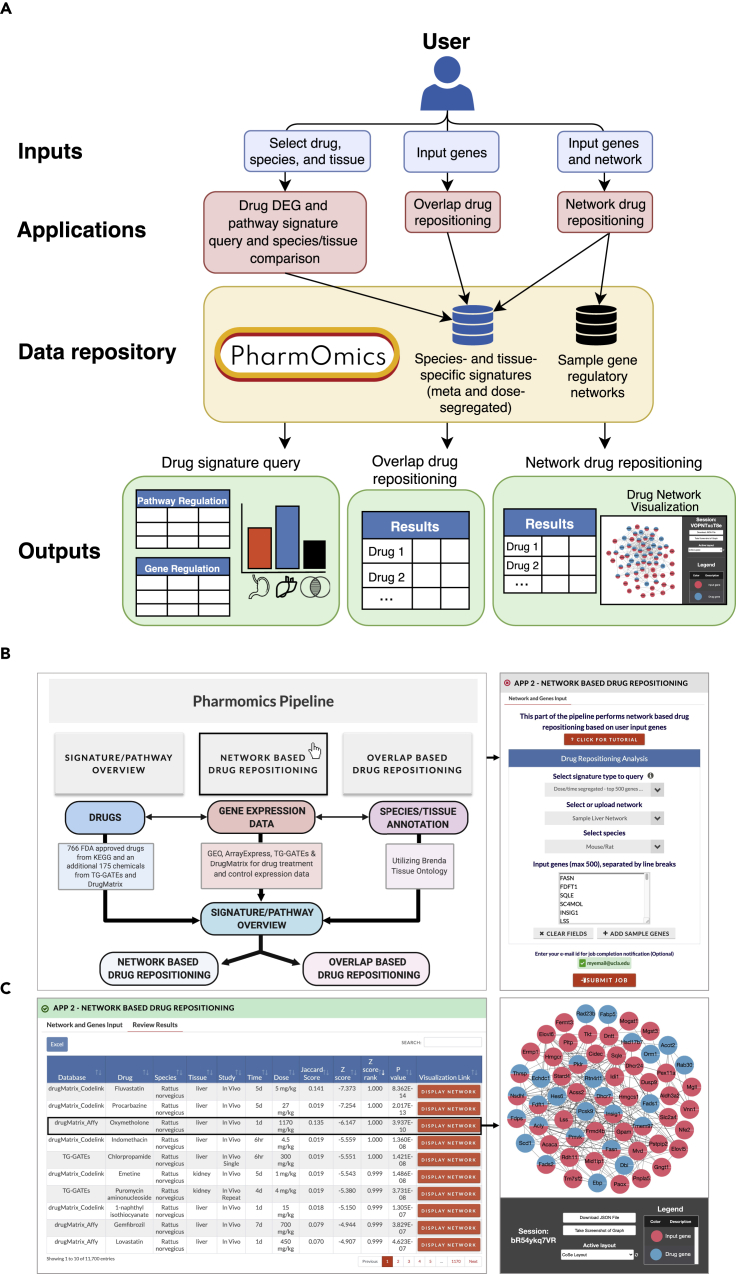
PharmOmics web server implementation (A) PharmOmics web server outline. The web server hosts drug signature and pathway queries, between-tissue and -species drug signature comparisons, and network-based and gene-overlap-based drug repositioning. Users can query, download, and perform drug repositioning using all species- and tissue-specific meta and dose-/time-segregated signatures. Interactive results tables and network visualizations are displayed on the website and available for download. (B) User interface of network drug repositioning web tool using a sample hyperlipidemia gene set and a sample mouse Bayesian gene regulatory network. Inputs to network drug repositioning includes (1) signature type to query (meta-analyzed, dose-/time-segregated with top 500 genes per signature, or dose-/time-segregated with all genes), (2) network (custom upload or select a sample network), (3) species (relating to the species of the network being used), and (4) genes. In the example case we choose dose-/time-segregated signatures using top 500 genes, a sample liver network, mouse/rat species, and the sample hyperlipidemia gene set (loaded from “Add sample genes”). If human gene symbols are provided with the “Mouse/Rat” species selection, the genes will be converted to mouse/rat symbols. (C) After the job is complete, the results file is displayed on the website and available for download. Subnetworks of top ranked drugs can be visualized using the “Display Network” button, which will load an interactive display of the subnetwork topology for a select drug. For example, the oxymetholone drug signature in rat liver is a top hit, and the drug network is shown on the right. Additional data in the downloadable results file include the genes that are both a drug gene and an input gene in the network, drug genes that are directly connected (first neighbor) to input genes, and input genes directly connected to drug genes.

To allow easy data access and use of the PharmOmics database, we provide drug signature query, species and tissue comparison, drug repositioning, and drug network visualization on an open access web server Mergeomics 2.0 (Shu et al., 2016; Ding et al., 2021) (http://mergeomics.research.idre.ucla.edu; STAR Methods). The PharmOmics web server features three main functions (Figure 2A). First, species- and tissue-stratified drug signatures and pathways for both the dose/time-segregated and meta signatures can be queried, and comparative analysis to examine similarities and differences between tissues and species for a given drug can be carried out. Second, it features a network drug repositioning tool that is based on the connectivity in a given gene network between PharmOmics drug signatures and user input genes such as a disease signature. Third, the web server offers a gene-overlap-based drug repositioning tool that assesses direct overlap between drug gene signatures and user input genes. The gene-overlap-based approach is similar to what has been previously implemented in other drug repositioning tools; however, the network-based repositioning approach is unique to PharmOmics. An example of network-based repositioning using a sample liver network and a sample hyperlipidemia gene set as inputs along with the resulting drug predictions and network visualization of a top drug, oxymetholone, are shown in Figures 2B and 2C. Lastly, network and gene overlap scores for hepatotoxicity and known ADR links from SIDER database are given in both network- and overlap-based analysis results to predict potential ADRs of the input signature.

### Utility of PharmOmics drug signatures in retrieving known therapeutic drugs for various diseases

Drug repositioning has mainly relied on analysis of direct overlaps between drug signatures and disease genes (Wang et al., 2016a, 2018; Subramanian et al., 2017). Recently, protein-protein interaction networks have also been used for network-based drug repositioning by assessing network connectivity between disease genes and known drug targets (Cheng et al., 2018). However, it remains unclear whether tissue-specific gene regulatory networks coupled with tissue-matched drug signatures are of value for drug repositioning. To this end, we evaluated the ability of PharmOmics drug signatures to identify drugs for diseases based on network connectivity of gene signatures of diseases and drugs matched by tissue in addition to the commonly used gene overlap approach. We hypothesized that if a drug is useful for treating a disease, the drug and disease signatures likely target similar pathways and therefore would have direct gene overlaps or connect extensively in gene networks. For network-based drug repositioning, we used a network proximity measure between drug DEGs and disease genes as previously described for protein-network-based analysis (Cheng et al., 2018) (STAR Methods). Here, we used tissue-specific Bayesian gene regulatory networks (BNs) and tested the mean shortest distance between drug DEGs and disease genes. For gene-overlap-based drug repositioning, we calculate the Jaccard score, gene overlap fold enrichment, and Fisher's exact test p values as measures of direct gene overlap.

**Figure 3:**
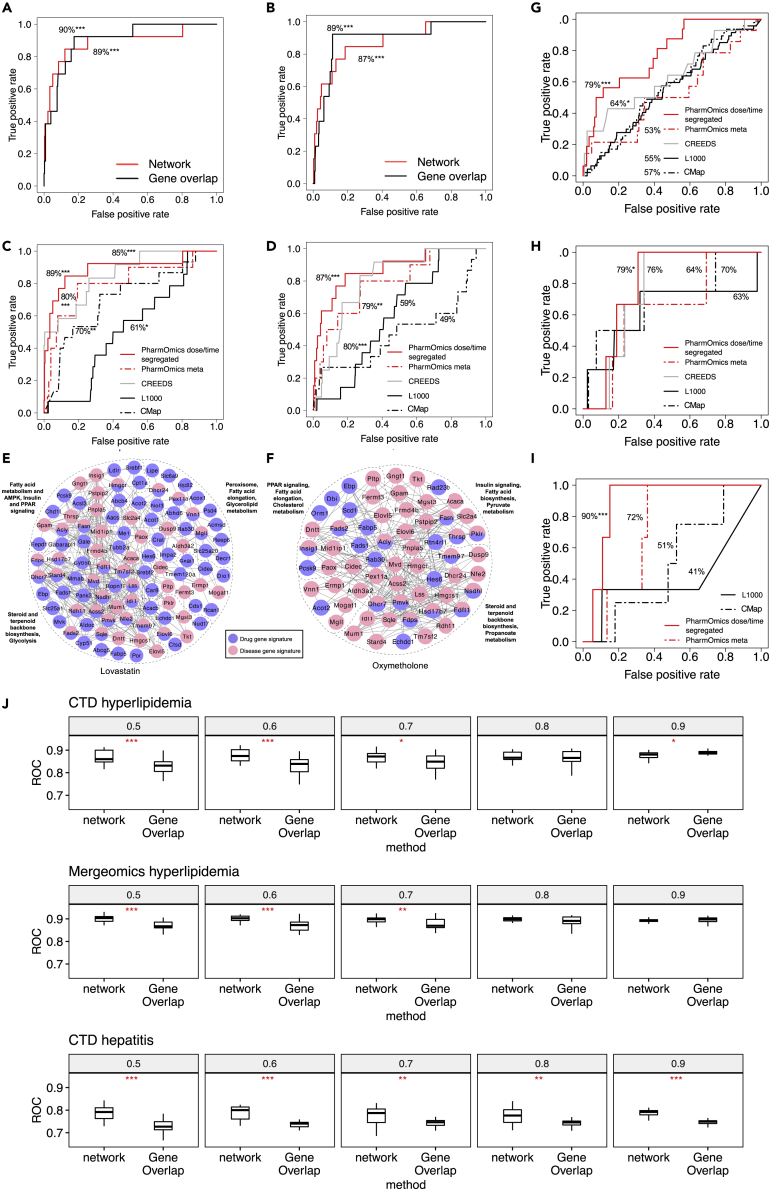
Drug repositioning using PharmOmics for diseases with known therapeutics (A and B) Area under the curve of receiver operating characteristics (AUROC) plots for network-based repositioning and gene-overlap-based repositioning in identifying antihyperlipidemia drugs (total n = 369, target n = 13) using (A) Mergeomics hyperlipidemia signature or (B) CTD hyperlipidemia signature. (C and D) Comparison of drug repositioning performance between PharmOmics network-based approach with CREEDS (total n = 281, target n = 12), using the “combined score” generated by the enrichment analysis tool implemented in Enrichr, L1000 (total n = 867, target n = 14), and CMap query system (total n = 934, target n = 15) using (C) Mergeomics hyperlipidemia signature and (D) CTD hyperlipidemia signature to identify antihyperlipidemic drugs. For drugs with multiple datasets with different doses and treatment times, only the best performing signature was used. (E and F) Drug-hyperlipidemia subnetwork based on Mergeomics hyperlipidemia signature (red) and drug signature (blue) showing first neighbor (direct) connections using (E) lovastatin and (F) oxymetholone signatures. Direct overlapping genes between disease and drug signatures are network nodes colored with both red and blue. (G–I) Comparison of drug repositioning performance between PharmOmics network-based approach with L1000, CREEDS, and CMap query system using CTD signatures for hepatitis (G), type 2 diabetes (H), and hyperuricemia (I) to identify steroid and nonsteroidal anti-inflammatory drugs (n = 16 in PharmOmics, n = 14 in CREEDS, n = 47 in CMap, n = 47 in L1000) (G), PPAR gamma agonists (n = 11 in PharmOmics, n = 9 in CREEDS, n = 13 in CMap, n = 13 in L1000) (H), and antihyperuricemia drugs (n = 3 in PharmOmics, n = 4 in CMap, n = 3 in L1000) (I), respectively. Note that in (I), CREEDS was not included due to lack of antihyperuricemic drugs. (J) Boxplot showing AUROC performance with different proportion of original disease signatures used after masking disease genes. For each proportion, 20 random sampling of original disease signature was conducted to obtain AUROC in identifying disease-related drugs. Wilcoxon signed rank test was used to calculate significance in difference between gene-overlap-based AUROC and network-based AUROC. ∗p < 0.05, ∗∗p < 0.01, and ∗∗∗p < 0.001, repectively, from Wilcoxon signed rank test. See also Tables S1–S3.

The performance of PharmOmics drug repositioning was first assessed using hyperlipidemia as the test case because multiple known drugs are available as positive controls. Because hyperlipidemia is most relevant to low-density lipoprotein cholesterol (LDL) and liver tissue, we retrieved LDL causal genes and pathways in liver tissue based on genome-wide association studies (GWAS) of LDL in conjunction with genetic regulation of liver gene expression using Mergeomics (STAR Methods) (Shu et al., 2016, 2017; Chella Krishnan et al., 2018). In addition to retrieving disease genes based on GWAS, a hyperlipidemia signature from CTD (Davis et al., 2017) was also used as an alternative disease signature source. For each drug with different dose and treatment durations, the signature with the highest overlap with the disease signature was used to represent the drug. Gene overlap- and network-based methods using dose-/time-segregated signatures had similar overall performance as assessed by the area under receiver operating characteristics (AUROC) curve (∼90% AUROC; p < 0.001) in the identification of antihyperlipidemic drugs (Figures 3A and 3B). The dose-/time-segregated signatures performed better than the meta signatures when using network-based repositioning (Figures 3C and 3D). When compared to other existing drug repositioning platforms, PharmOmics was able to retrieve higher prediction rankings for the known antihyperlipidemia drugs (Table 1) than CMap (Mergeomics signature p = 0.0064, CTD signature p = 0.0056) and L1000 (Mergeomics signature p = 0.03, CTD signature p < 0.001), while showing comparable results to CREEDS (non-significant for both Mergeomics and CTD signatures) based on Wilcoxon signed rank test. PharmOmics also reached better AUROC (Figures 3C and 3D) than CMap and L1000, as well as higher balanced accuracy, defined as (sensitivity + specificity)/2 (Table 2), than CREEDS, CMap, and L1000. These results support the capacity of PharmOmics as a complementary drug repositioning tool to existing platforms.

**Table 1:** Prediction percentile of FDA-approved antihyperlipidemic drugs based on hyperlipidemia signatures from Mergeomics (MO) pipeline and CTD database across different platforms tested

Platform	PharmOmics dose-/time-seg network		PharmOmics dose-/time-seg Jaccard		PharmOmics meta		CREEDS		CMap		CMap HEPG2		L1000		L1000 HEPG2	
Disease gene signature	MO	CTD	MO	CTD	MO	CTD	MO	CTD	MO	CTD	MO	CTD	MO	CTD	MO	CTD
Atorvastatin	0.951	0.794	0.981	0.957	0.498	0.316	0.989	0.82	0.913	0.164	0.414	0.31	0.962	0.668	0.405	0.307
Bezafibrate	0.856	0.995	0.901	0.982	0.981	0.932	0.571	0.95	0.332	0.561	0.439	0.915	0.394	0.755	NA	NA
Cerivastatin	0.989	0.848	0.995	0.962	0.798	0.719	0.986	0.836	0.879	0.516	NA	NA	0.967	0.761	NA	NA
Clofibrate	0.965	0.97	0.802	0.927	0.951	0.992	0.737	0.986	0.196	0.291	0.153	0.433	0.31	0.615	NA	NA
Clofibric acid	0.93	0.58	0.949	0.892	NA	NA	NA	NA	NA	NA	NA	NA	NA	NA	NA	NA
Fenofibrate	0.984	0.986	0.908	0.883	0.954	0.954	0.797	0.943	0.121	0.108	0.229	0.201	NA	NA	NA	NA
Fluvastatin	1	0.997	1.000	0.924	0.97	0.985	1	0.815	0.905	0.963	0.807	0.118	0.958	0.514	0.513	0.327
Gemfibrozil	0.992	0.962	0.984	0.873	0.787	0.844	0.9	0.712	0.677	0.612	NA	NA	0.363	0.591	NA	NA
Lovastatin	0.995	0.984	0.986	0.986	0.905	0.43	0.993	0.632	0.972	0.084	0.528	0.346	0.992	0.979	0.415	0.765
Nafenopin	0.726	0.943	0.472	0.864	NA	NA	0.431	0.712	NA	NA	NA	NA	NA	NA	NA	NA
Niacin	0.192	0.873	0.821	0.309	0.137	0.711	0.719	0.343	0.671	0.171	0.606	0.069	0.107	0.307	NA	NA
Pravastatin	0.894	0.339	0.911	0.862	NA	NA	0.979	0.854	0.829	0.669	0.727	0.934	0.592	0.717	NA	NA
Simvastatin	0.949	0.935	0.856	0.992	0.916	0.909	0.996	0.9	0.972	0.951	0.844	0.573	0.987	0.843	0.595	0.425
Ciprofibrate	NA	NA	NA	NA	NA	NA	NA	NA	0.685	0.998	0.84	0.288	0.292	0.272	NA	NA
Ezetimibe	NA	NA	NA	NA	NA	NA	NA	NA	0.905	0.982	0.514	0.757	0.657	0.269	0.366	0.101
Probucol	NA	NA	NA	NA	NA	NA	NA	NA	0.552	0.115	0.021	0.696	0.018	0.529	NA	NA
Rosuvastatin	NA	NA	NA	NA	NA	NA	NA	NA	0.913	0.056	0.855	0.238	0.905	0.464	NA	NA
Median	0.951	0.943	0.911	0.924	0.911	0.876	0.94	0.828	0.829	0.516	0.528	0.346	0.624	0.603	0.415	0.327
Mean	0.879	0.862	0.890	0.878	0.79	0.779	0.841	0.792	0.701	0.483	0.537	0.452	0.607	0.592	0.459	0.385
Total number of drugs	369	369	369	369	263	263	281	281	934	934	667	667	867	867	153	153

HEPG2 results from both L1000 and CMap were retrieved for tissue specificity comparison.

**Table 2:** Comparison of drug repositioning performance between PharmOmics and other existing platforms for hyperlipidemia

Drug signature platform	Total FDA-listed drugs	Significant drugs (% total)	Known hyperlipidemia drugs	Significant hyperlipidemia drugs (% known drugs)	Balanced accuracy **(sensitivity + specificity/2)**
PharmOmics meta_liver	263	33 (12.5%)	10	6 (60%)	74.7%
PharmOmics dose/time segregated_liver - network	369	29 (7.9%)	13	9 (69.2%)	81.8%
PharmOmics dose/time segregated_liver - Jaccard	369	171 (46.3%)	13	12 (92.3%)	73.8%
CMap	934	264 (28.6%)	15	8 (53.3%)	62.7%
CMap_HEPG2	667	135 (20.3%)	13	1 (7.7%)	43.6%
L1000	867	428 (49.3%)	14	8 (57.1%)	54.1%
L1000_HEPG2	153	37 (24.2%)	5	0 (0%)	37.5%
CREEDS_liver	281	257 (91.4%)	12	12 (100%)	54.5%

Drug pool for each database was limited to FDA-approved drugs to match the drug selection criteria in PharmOmics to make results comparable. Significance were defined at the recommended cutoffs for each platform: z-score < −2.33 in PharmOmics, overlap BH adjusted p < 0.05 in CREEDS, L1000, and PharmOmics dose-/time-segregated Jaccard, and connection score >95 or < −95 in CMap query system. For CMap and L1000, drug signatures from all cell lines (CMap_all or L1000_all) or from the hyperlipidemia relevant liver cell line HepG2 (CMap_HEPG2 or L1000_HEPG2) were used.

To provide molecular insights into the top drug predictions, we examined the disease network overlap patterns of the top drugs, lovastatin, a known antihyperlipidemia drug (Figure 3E), and oxymetholone, a known androgen drug with hyperlipidemia ADR (FDA box warning label) (Figure 3F). The network approach can retrieve both therapeutic drugs and drugs with ADRs because network connectivity rather than direction of change was the main consideration. Both drugs had DEGs connecting to genes related to cholesterol metabolism and peroxisome proliferator-activated receptor (PPAR) pathways in the hyperlipidemia network (Figures 3E and 3F). However, lovastatin DEGs had direct overlap with cholesterol biosynthesis genes such as *Hmgcr* (target of statin drugs) and *Sqle* along with more DEGs that connected to disease genes, whereas oxymetholone did not have *Hmgcr* and *Sqle* as DEGs and had smaller disease subnetwork overlap, suggesting key differences between the two drugs. Notably, many drug DEGs did not directly overlap with disease genes, which supports the utility of a network-based drug repositioning approach that does not require the direct retrieval of a known drug target or direct overlap of drug DEGs with disease genes.

We further evaluated the performance of PharmOmics in retrieving known drugs for several other diseases for which known therapeutic drugs are available and can serve as positive controls. Using CTD disease signatures for hepatitis, network-based repositioning obtained 79% AUROC (p < 0.001, Figure 3G) in retrieving both steroid and nonsteroid anti-inflammatory agents (prediction ranks in Table S1). We also queried type 2 diabetes signatures and found PharmOmics was able to predict PPAR gamma agonist drugs (79% AUROC, p = 0.04, Figure 3H), but not sulfonylurea drugs that act on the pancreatic islets to enhance insulin release (prediction ranks in Table S2), due to a paucity of drug signatures in islets. Finally, we queried hyperuricemia signatures and network-based repositioning obtained 90% AUROC (p = 0.009, Figure 3I, prediction ranks in Table S3) for detecting antihyperuricemia drugs. The overall performance of PharmOmics for these various diseases is better or on par with other platforms (Figures 3G–3I).

We reasoned that network-based repositioning is likely more robust against missing genes in disease signatures than traditional gene-overlap-based analysis. To test this, we masked part of the disease gene signatures for hyperlipidemia and hepatitis as test cases. Results showed that network-based repositioning maintained similar performance even when 50% of disease genes were masked, whereas gene-overlap-based strategy showed a decrease in performance when 20% or more genes were masked from the disease signatures (Figure 3J).

Overall, these various test cases using known therapeutic drugs as positive controls support both the utility and robustness of network-based drug repositioning for the diseases tested when drug signatures from the appropriate tissues are available.

### Utility of PharmOmics to predict known and novel drugs for nonalcoholic fatty liver disease

After establishing the performance of PharmOmics in drug repositioning using the aforementioned case studies where positive controls are available, we applied PharmOmics to predict potential drugs for nonalcoholic fatty liver disease (NAFLD), for which there is currently no approved drugs. Using NAFLD steatosis signatures from a published mouse study (Chella Krishnan et al., 2018) and the CTD NAFLD signatures (Davis et al., 2017), we predicted PPAR alpha agonists (clofibrate, fenofibrate, bezafibrate, and gemfibrozil), HMG-CoA reductase inhibitors (lovastatin, fluvastatin, and simvastatin), a PPAR gamma agonist (rosiglitazone), and a nonsteroidal anti-inflammatory drug (aspirin) to be among the top 10% of drug candidates based on the average ranking of drugs predicted using both the mouse steatosis signature and CTD NAFLD signature (Table S4). PPAR agonists have been well supported as potential drugs for NAFLD (Laurin et al., 1996; Hertz and Bar-Tana, 1998; Neuschwander-Tetri et al., 2003; Kawaguchi et al., 2004; Fernández-Miranda et al., 2008; Ratziu et al., 2008, 2010; Nan et al., 2009; Torres et al., 2011; Zhang et al., 2015; Abd El-Haleim et al., 2016; Liss and Finck, 2017), whereas statins showed positive association yet less literature documentation (Pastori et al., 2015; Park et al., 2016; Sigler et al., 2018; Bravo et al., 2019). Aspirin was recently reported to be associated with reducing liver fibrosis progression in a cohort association study in humans (Simon et al., 2019), but here it was predicted for liver steatosis or general NAFLD.

Next, we sought to validate the top predicted drugs in mitigating liver steatosis. We chose fluvastatin as a positive control due to its high prediction rank across different platforms (top 5% in PharmOmics, CMap, and L1000; top 20% in CREEDS; Table S4) and better efficacy compared with other statins in improving metabolic phenotypes in a methionine- and choline-deficient diet mouse model used to study nonalcoholic steatohepatitis (NASH) (Park et al., 2016). We also chose to test aspirin as a unique top prediction by PharmOmics (top 5%). In comparison, aspirin had much lower ranks in CREEDS (30%) and CMap (35%) and was not documented in L1000.

**Figure 4:**
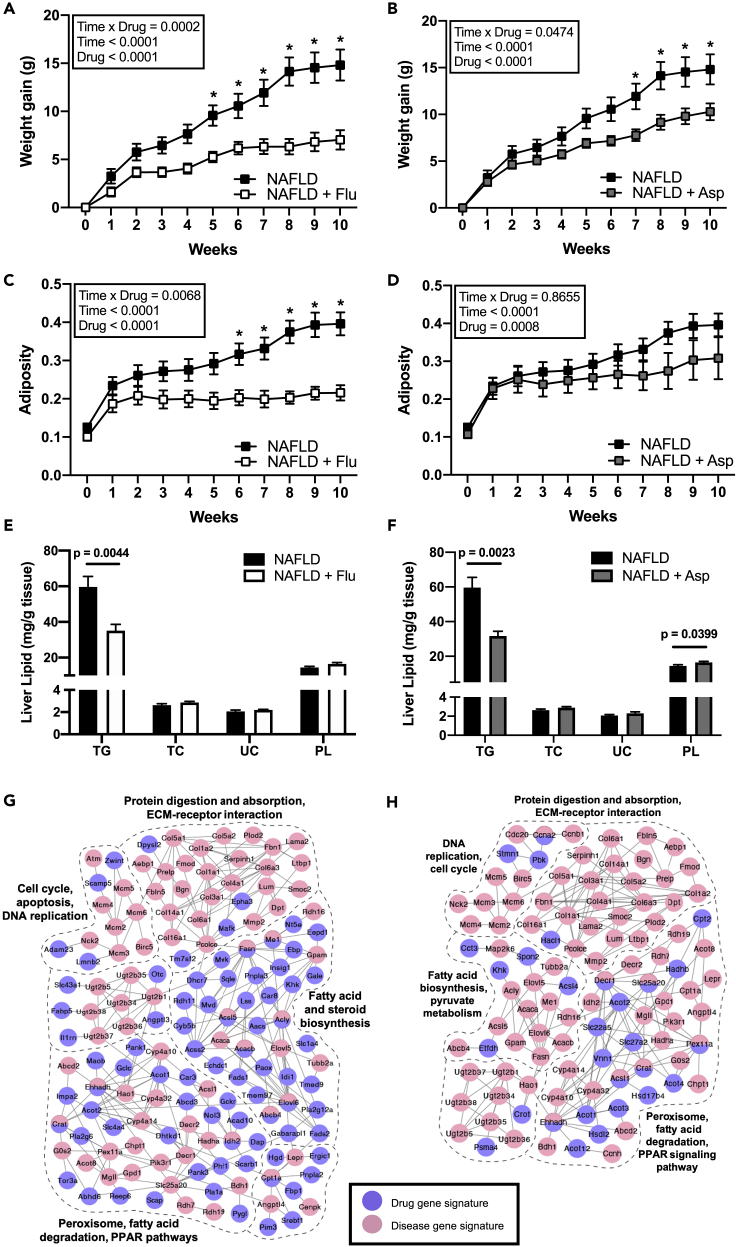
*In vivo* validation of top predicted drugs fluvastatin and aspirin on preventing NAFLD phenotypes in a diet-induced NAFLD mouse model Mouse groups include C57BL/6J mice fed a high-fat high-sucrose (HFHS) diet to induced liver steatosis (NAFLD), HFHS with fluvastatin (NAFLD + Flu), and HFHS with aspirin (NAFLD + Asp). (A and B) Time course of body weight gain in NAFLD mice treated with fluvastatin (A) or aspirin (B) over 10 weeks. (C and D) Time course of fat mass and muscle mass ratio (adiposity) in mice treated with fluvastatin (C) or aspirin (D) over 10 weeks. (A–D) Data are represented as mean ± SEM and were analyzed by two-way ANOVA followed by Sidak post-hoc analysis to examine treatment effects at individual time points. p <0.05 was considered significant and is denoted by an asterisk (∗). (E and F) Quantification of lipids in the liver of mice on fluvastatin (E) or aspirin (F) treatment for 10 weeks. Triglyceride (TG), total cholesterol (TC), unesterified cholesterol (UC), phospholipid (PL). Data are represented as mean ± SEM and were analyzed using two-sided t test. p <0.05 was considered significant and is denoted by an asterisk (∗). (A-F) Sample size n = 7–8/group. (G) Gene network view of fluvastatin gene signatures overlapping with NAFLD disease signatures. (H) Gene network view of aspirin gene signatures overlapping with NAFLD disease signatures. See also Figures S1 and S2; Table S4.

Fluvastatin and aspirin were tested using a mouse steatosis model induced by a high-fat high-sucrose (HFHS) Western diet, which has been previously used to study NAFLD (Hui et al., 2015; Chella Krishnan et al., 2018, 2021; Norheim et al., 2021). Key genes identified in this diet-induced NAFLD model (Chella Krishnan et al., 2018) were known NAFLD-associated genes (Chakravarthy et al., 2009; Wu et al., 2013) and reproducible in independent human studies (Lee et al., 2017), supporting its utility as a model for this disease. Comparison between the mice in HFHS group (NAFLD) and the chow group (Control) confirmed that HFHS induced increases in hepatic triglycerides (TG), a measure of liver steatosis, without significant differences in liver weight or other lipids (Figure S1). Comparison of the fluvastatin- and aspirin-treated groups with the NAFLD group revealed significant treatment effects by both drugs on mitigating body weight gain (fluvastatin: p < 0.0001, Figure 4A; aspirin: p < 0.0001, Figure 4B), reducing adiposity (fluvastatin: p < 0.0001, Figure 4C; aspirin: p = 0.0008, Figure 4D), and decreasing hepatic triglycerides (TG) (fluvastatin: p = 0.0044, Figure 4E; aspirin: p = 0.0023, Figure 4F). Drug treatments did not significantly alter liver weight, total cholesterol (TC), and unesterified cholesterol (UC) (Figures 4E, 4F, and S2).

We further investigated whether the effects of the drugs on NAFLD steatosis phenotypes were confounded by food and water intake. No difference was observed in food and water intake in the fluvastatin treatment group (Figures S2E and S2F), but in the aspirin treatment group there was a significant decrease in food intake but no difference in water intake compared with the NAFLD group (Figures S2G and S2H). Adjusting for food intake effects using linear regression showed that the effects of fluvastatin on body weight gain (p = 0.0306), adiposity (p = 0.0022), and hepatic TG (p = 0.0190) remained significant. For aspirin, the significant effects on hepatic TG (p = 0.0372) remained, but the effects on body weight gain and adiposity (p = 0.0511) were no longer significant.

Our experimental validation experiments support the efficacy of both fluvastatin and aspirin in mitigating liver TG levels independent of food and water intake. Agreeing with the PharmOmics prediction ranks, the effects of fluvastatin were stronger than that of aspirin (Figures 4A–4F). Moreover, visualization of the network overlaps between NAFLD signatures and drug signatures revealed more extensive disease network connections for fluvastatin than for aspirin (Figures 4G and 4H), and the signatures of the two drugs connected to pathways involved in NAFLD such as PPAR signaling pathways and fatty acid and steroid biosynthesis.

### Utility of PharmOmics drug signatures in predicting and understanding hepatotoxicity

We further explored the potential of coupling PharmOmics drug signatures and tissue gene networks to predict liver toxicity, a major type of ADR for which both toxicity signatures and orthogonal ADR documentations from various independent databases are available for performance evaluation. We used the chemical-induced liver injury signature containing 435 genes from CTD to predict the degree of hepatotoxicity of drugs based on the overlap and liver gene network connectivity between PharmOmics drug signatures and the CTD liver injury signature. We then used both the liver histological severity from TG-GATEs and the independent FDA drug-induced liver injury (DILI) categories (“most”, “less”-moderate/mild, and “no” DILI concern) as *in silico* independent validation of the predicted hepatotoxic drugs.

**Figure 5:**
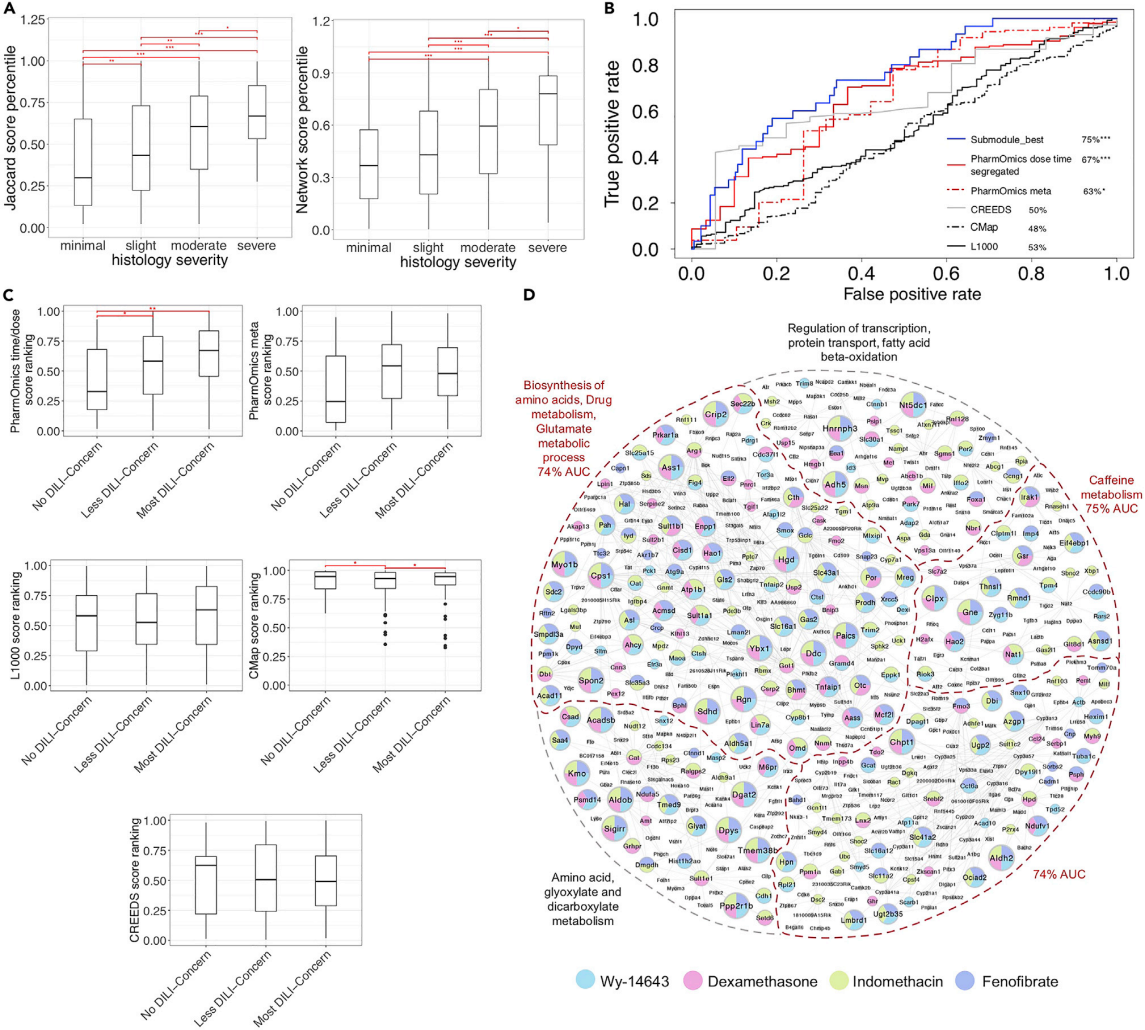
Utility of PharmOmics drug signatures in hepatotoxicity prediction The analysis was based on matching between PharmOmics drug signatures and hepatotoxicity signatures of drug-induced liver injury (DILI) curated from comparative toxicogenomics database (CTD). (A) Boxplots of gene-overlap-based hepatotoxicity ranking (left) and network-based hepatoxicity ranking (right) by PharmOmics, across four categories of liver injury histological severity defined by the independent TG-GATEs database (x axis) (all doses included, n = 205 in “minimal” category, n = 221 in “slight” category, n = 147 in “moderate” category, n = 37 in “severe” category). (B) ROC curves comparing PharmOmics with other tools in predicting hepatotoxic drugs from the FDA DILI drug database. For PharmOmics, three sets of tests were performed, where dose/time-segregated drug signatures, meta signatures, or a hepatotoxicity subnetwork was used. Significance were calculated by comparing “no DILI-concern” category (n = 30 in PharmOmics dose/time segregated signatures, n = 19 in PharmOmics meta, signatures, n = 94 in CMap, n = 88 in L1000, n = 18 in CREEDS) versus “less DILI-concern” plus “most DILI-concern” categories (n = 185 in PharmOmics dose/time segregated signatures, n = 156 in PharmOmics meta signatures, n = 276 in CMap, n = 251 in L1000, n = 142 in CREEDS). (C) Hepatotoxicity signature matching scores from various drug repositioning platforms across three different DILI drug categories. For drugs with multiple dose and time points, only the best score was used. PharmOmics scores are derived from network-based matching; CMap scores were derived from the CMap online query plarform; L1000 scores are from Jaccard scores from the L1000 plarform; CREEDS scores are from the combined scores derived from the enrichr platform. Boxplots in A and C show IQR (IQR) and median values (line inside the box). IQR was defined as between 25th (Q1) and 75th (Q3) percentile. The upper and lower bars indicate the points within Q3 + 1.5∗IQR and Q1 – 1.5∗IQR, respectively. (D) Liver hepatotoxicity network based on CTD hepatotoxicity genes and its overlap with drug signatures of four of the top five predicted drugs by PharmOmics that had >50 drug signature genes. Phenobarbital was among the top five drugs but was not included in the figure due to its small DEG size. Colors of the network nodes denote the different drugs targeting the genes. The top three predictive subnetworks are labeled in red. Kruskal-Wallis test followed by post-hoc pairwise Wilcoxon signed rank test was used for statistics in A and C, and Wilcoxon signed rank test was used to calculate significance for B ∗p < 0.05, ∗∗p < 0.01, and ∗∗∗p < 0.001, respectively. See also Table S5.

We found that drug ranking of hepatotoxicity from both the network-based and gene overlap-based analyses from PharmOmics increased with higher histological severity as defined by TG-GATEs (Figure 5A), supporting a positive relationship between the predicted hepatotoxicity scores and experimental hepatotoxicity measures. Next, we tested the performance of PharmOmics in predicting hepatotoxic drugs from the FDA DILI drug database. PharmOmics dose-/time-segregated signatures resulted in higher performance (67% AUROC, p = 0.0014) compared with the meta signatures (63% AUROC, p = 0.029) and the other platforms tested such as CREEDS, CMap, and L1000 (AUROC 48–53%; nonsignificant p > 0.05 for CREEDS and L1000; CMap showed significantly higher scores in drugs with lower hepatotoxicity, Figures 5B and 5C).

Top drug predictions by PharmOmics based on the CTD hepatotoxicity signatures were wy-14643 (experimental drug with severe histological finding in TG-GATEs), dexamethasone (moderate DILI concern category in FDA and moderate histological finding in TG-GATEs), phenobarbital (moderate DILI concern), indomethacin (“most” DILI concern), and fenofibrate (moderate DILI concern). The network overlapping patterns of the top predicted drugs with the CTD liver injury genes (Figure 5D) showed that the top predicted drugs exhibited consistent targeting of the hepatotoxicity gene subnetworks.

Because CTD contains a large number (435) of curated hepatotoxicity genes, we hypothesized that this large network could be divided into subnetworks indicative of different mechanisms toward liver toxicity, which might improve toxicity prediction for drugs with different mechanisms. Therefore, we applied the Louvain clustering method to divide the liver injury network defined by the CTD hepatotoxicity genes into subnetworks and filtered out subnetworks with less than 10 genes. These subnetworks showed varying abilities in identifying drugs with DILI concerns (Table S5). The best performing hepatotoxicity subnetwork showed improved AUROC compared with the whole network (75 vs. 67%; Figure 5B). Further scrutinization of the top performing subnetwork revealed that the antioxidant gene *GSR*, the phase 2 drug metabolizer *NAT2*, and the inflammatory response gene *IRAK1* showed the best predictability. These results suggest that the network-based toxicity prediction approach may help prioritize predictive genes, pathways, and subnetworks related to hepatotoxicity.

### Utility of meta signatures to understand tissue and species specificity

**Figure 6:**
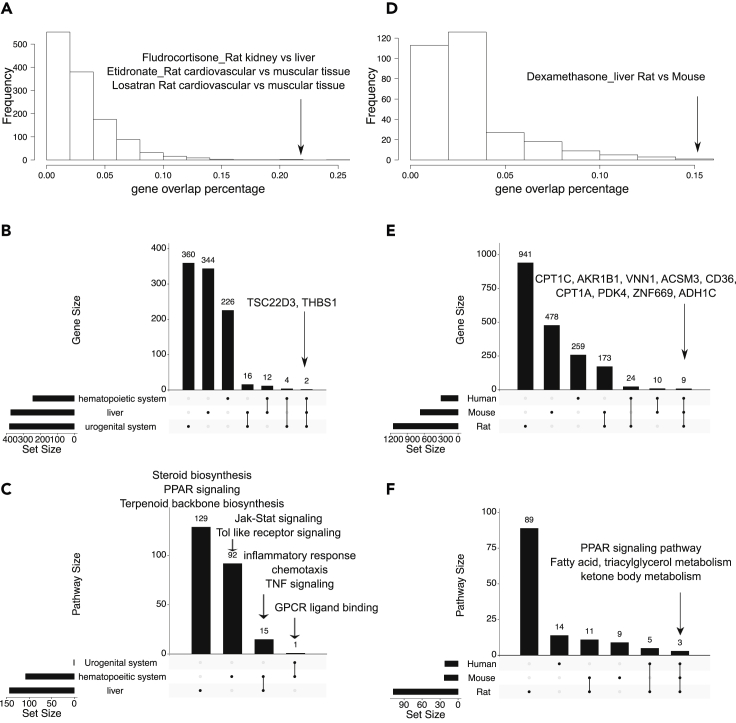
Cross-tissue and cross-species comparisons of drug signatures in PharmOmics (A) Distribution of drug signature overlap percentages between tissue pairs in matching species from PharmOmics meta signature database. Arrow points to the pairs of tissues for drugs with high overlap in gene signatures. (B) Upset plot of cross-tissue comparison for atorvastatin signatures genes. Y axis indicates number of genes. (C) Upset plot of cross-tissue comparison for pathways enriched in atorvastatin signatures. Y axis indicates number of pathways. (D) Distribution of drug signature overlap percentages between species pairs for matching tissues from PharmOmics meta signature database. Arrow points to the species pair with high gene signature overlap for a matching drug. (E) Upset plot of cross-species comparison for rosiglitazone liver gene signatures. Y axis indicates number of genes. (F) Upset plot of cross-species comparison for pathways enriched in rosiglitazone liver signatures. Y axis indicates number of pathways. Pairs of tissues or species with shared drug signature genes or pathways are connected with black vertical lines in the bottom portion of the Upset plots in B, C, E and F. See also Tables S6 and S7.

To evaluate tissue and species specificity of drug signatures, we used the meta signatures, which reflect the dose-/time-independent, consistent genes affected by drugs across studies in the same tissue or species. We analyzed the overlap in gene signatures for each drug across different tissues and species and visualized the results using UpSetR (Conway et al., 2017). As shown in Figure 6A, the overlap rate in the DEGs of the same drug between tissues and organs is usually less than 5%, indicating a high variability in DEGs between tissues.

As an example, we examined atorvastatin, an HMGCR (β-Hydroxy β-methylglutaryl-CoA receptor) inhibitor, which has well understood mechanisms and has been broadly tested in different tissues under the human species label. We found that two DEGs, *TSC22D3* and *THBS1*, involved in extracellular matrix and inflammation, respectively, were shared across tissues (Figure 6B). At the pathway level, immune-related pathways were shared between blood and liver cells but not in the urogenital system (Figure 6C; Table S6). Unique liver pathways include steroid synthesis and drug metabolism, which is expected as the known target of statin drugs is HMGCR, the rate limiting enzyme in cholesterol biosynthesis in liver. Blood monocyte DEGs indicated changes in inflammation-related pathways, whereas G-protein-coupled receptor (GPCR) ligand binding proteins were altered in prostate cancer cells. The tissue specificity of drug meta signatures supports tissue-specific therapeutic responses and emphasizes the need for comprehensive inclusion of drug signatures from different tissue systems.

We also found evidence for high species specificity. As shown in Figure 6D, the pairwise overlaps in DEGs between species for the same drug is generally lower than 5%. Here we chose PPAR gamma receptor agonist rosiglitazone as an example because this drug has datasets across human, rat, and mouse in PharmOmics, and its mode of action is well studied. As shown in Figures 6E and 6F, nine genes (*CPT1C*, *AKR1B1*, *VNN1*, *ACSM3*, *CD36*, *CPT1A*, *PDK4*, *ZNF669*, *ADH1C*) and several pathways (PPAR signaling and fatty acid, triacylglycerol, and ketone body metabolism) were consistently identified from liver DEGs across species (Table S7), reflecting the major species-independent pharmacological effects of rosiglitazone. Bile-acid related genes were altered in rat datasets, whereas retinol metabolism and adipocytokine pathways were altered in human datasets. The species differences identified highlight the importance of investigating the physiological differences among model systems to facilitate drug design with better translational potential. Our cross-species comparative analysis also revealed that only 21% of the unique drug-tissue pairs (236 out of 1,144) have data from two or more species, thus highlighting the need for systematic data generation across species to better understand between-species similarities and differences in drug actions.

## Discussion

We present PharmOmics, an open-access drug signature database along with a web interface for accessing and utilizing the signatures for various applications. PharmOmics utilizes publicly available drug-related transcriptomic datasets across multiple data repositories and provides unique tissue-, species-, and dose-/time-stratified gene signatures that are more reflective of *in vivo* activities of drugs. We also developed a unique framework for drug repositioning based on tissue-specific gene network models. We examined the potential applications of PharmOmics for various utilities including drug repurposing, toxicity prediction, and comparison of molecular activities between tissues and species. We also carried out *in silico* performance assessments across drug signature databases and *in vivo* mouse experiments to validate select drugs from network-based predictions for liver steatosis.

Compared with the well-established CMap and LINC1000 platforms, PharmOmics focuses more on *in vivo* settings and likely captures physiologically relevant drug signatures to improve drug repositioning performance. Compared with a previous crowdsourcing effort that also utilizes publicly available drug datasets (Wang et al., 2016a), the PharmOmics platform includes more curated databases (TG-GATEs, DrugMatrix Affymetrix, DrugMatrix Codelink datasets) and has a systematic tissue, species, and treatment regimen stratification to facilitate drug repositioning. Comparison across platforms revealed statistically significant gene signature overlaps, but the degree of overlap is low (Figure S3), supporting that these are complementary platforms. PharmOmics is also the only tool utilizing a gene network framework rather than a direct gene overlap approach. We believe that the increased coverage of *in vivo* datasets; consideration of tissue-, species-, and dose specificity; and the use of a network approach all contribute to the improved performance of PharmOmics. However, in cases where tissues, networks, and doses are not available in PharmOmics, existing platforms have advantages.

The use of tissue annotation with BRENDA Tissue Ontology helps normalize organ labels and improves comparability of datasets. The tissue- and species-specific analyses implemented in PharmOmics allows for comprehensive molecular insight into the actions of drug molecules in individual tissues and species. Our results support that different species have unique drug responses in addition to shared features. Therefore, drug responses obtained in animal models require caution when translating to humans. This notion agrees with the long-observed high failure rate of drug development that has primarily relied on preclinical animal models and argues for greater consideration and understanding of inter-species differences in drug actions.

In addition to tissue and species stratification, we also provide detailed dose-/time-segregated drug signatures, which can help better understand the dose- and time-dependent effects of drugs through gene signature and pathway comparisons offered through our web server. By contrast, the meta-analysis signatures capture the consistent genes and pathways across treatment regimens, which likely represent core, dose-/time-independent mechanisms, and can help address the sample size issue of individual datasets, as most drug treatment datasets carried out to date are of small sample size. Repositioning with meta signatures also significantly shortens the computing time in network-based repositioning applications. For instance, computation using 1,251 human meta signatures can be completed in 40 min, whereas using ∼14,000 dose-/time-segregated signatures can take 4 h.

Previous drug repositioning studies support the utility of a protein-network-based approach for drug repositioning. Here we show that combining the drug transcriptomic signatures in PharmOmics with tissue-specific gene regulatory networks and gene signatures of diseases can predict potential therapeutic avenues and tissue toxicity. Compared with other platforms, the use of tissue- and species-specific drug signatures along with network biology is a unique feature of PharmOmics, which enables drug prioritization based on network proximity rather than direct gene overlaps. We demonstrate in various applications that network-based analysis had more robust performance to that of gene overlap-based analysis. Moreover, network-based repositioning offers molecular and mechanistic insights into the therapeutic or toxic effects of drugs. For instance, different NAFLD network overlapping patterns were observed between fluvastatin and aspirin, which reflect different drug mechanisms for the same disease phenotype that can be explored further.

In conclusion, we have established a new drug signature database, PharmOmics, across different dosage, species, and tissues, which captures the systems level *in vivo* activities of drug molecules. In addition, we demonstrate the possible means to integrate these signatures with network biology to address drug repositioning needs for disease treatment and to predict and characterize toxicity. Finally, our study tested the concept of tissue-matched drug repositioning and supports consideration of the tissue context of disease and drugs in the improvement of drug repositioning performance, and repositioning efforts will be further expanded when more tissue-specific disease and drug signatures are available. PharmOmics has the potential to complement other available drug signature databases to accelerate drug development and toxicology research. It should be noted that we aim to position PharmOmics as a data-driven tool for hypothesis generation. Integration with known drug characteristics to select drug candidates and conducting follow-up experiments are still essential.

### Limitations of the study

There are several limitations in this study. First, our computational pipeline may not be able to identify all drug datasets from GEO and ArrayExpress database and currently does not accommodate RNA sequencing datasets (∼10% of retrieved drug datasets). Variations in annotations of drug names, sample size, definition of treatment versus control groups, and tissue/cell line labeling across datasets make it challenging to design a fully automated pipeline to curate drug datasets. Another issue is that deposited RNA sequencing datasets are in nonstandardized formats, with some as raw counts and others as normalized counts such as FPKM and RPKM, making a streamlined and standardized analysis of these datasets difficult. We are currently processing RNA sequencing datasets and will add these to PharmOmics in the future. It is therefore crucial for public data repositories to offer clear definitions and instructions for metadata generation in order to standardize terms and data processing procedures across datasets to facilitate future data reuse. Secondly, the coverage of tissue, species, and treatment regimens across drugs is unbalanced, preventing a thorough comparison across tissues, species, dosages, and treatment windows. We will continue to update our PharmOmics database periodically to include more datasets as they become available to increase the coverage of datasets and drug signatures. Thirdly, the sample sizes for drug treatment studies tend to be small (majority with n = 3/group or less). This is an intrinsic limitation of existing drug studies and is a common challenge to existing drug databases including TG-GATEs, DrugMatrix, CMap, L1000, and CREEDS. This fact highlights the need for systematic efforts to increase sample sizes in drug genomic studies. To mitigate the sample size concern and reduce the reliance on individual studies, we implemented a meta-analysis strategy to aggregate drug signatures across studies to derive meta signatures. However, this strategy removes dosage- and time-dependent effects. We offer both options in our database to mitigate sample size concerns through meta-analysis while retaining dose and time regimen information through the dose/time-segregated analysis. Fourth, our network-based applications are currently limited in the coverage of high-quality tissue specific regulatory networks and computing power. We will continue to expand and improve the tissue networks and computing environment in our web server. Lastly, systematic validation efforts are needed to substantiate the value of drug repositioning tools such as PharmOmics. Thus far, we utilized both *in silico* performance assessments and *in vivo* experiments to validate our predictions in limited settings. As with the other existing platforms such as CMap and L1000, future application studies and community-based validation efforts are necessary to further assess the value of PharmOmics.

## STAR★Methods

### Key resources table

**Table:** 

REAGENT or RESOURCE	SOURCE	IDENTIFIER
**Chemicals, peptides, and recombinant proteins**		

Aspirin	Cayman Chemicals	Cat# 70260
Fluvastatin (sodium salt)	Cayman Chemicals	Cat# 10010337
High fat high sucrose Diet (Purified Rodent Diet to Match Condensed Milk Diet)	Research diets	D12266B
Triton X-100	Sigma-Aldrich	Cat# T8532-500ML
Chow diet (Lab Rodent Diet 5053)	Lab Diet (From the UCLA animal husbandry)	

**Critical commercial assays**		

Triglyceride assay (analyzed by the UCLA GTM Mouse Transfer Core)	Sigma-Aldrich	Cat# TR0100-1KT
Cholesterol assay (analyzed by the UCLA GTM Mouse Transfer Core)	UCLA GTM Mouse Transfer Core	NA
Phospholipids C kit assay (analyzed by the UCLA GTM Mouse Transfer Core)	Wako Diagnostics	Cat# 997-01801

**Deposited data**		

Code for drug repositioning	This paper	https://github.com/XiaYangLabOrg/pharmomics
Meta-analysis based and dose/time segregated Drug signatures	This paper	http://mergeomics.research.idre.ucla.edu/runpharmomics.php

**Experimental models: Organisms/strains**		

C57BL/6J, male, 7 weeks old	The Jackson Laboratory	#000664

**Software and algorithms**		

R 4.0.2	R Core Team	https://www.r-project.org/
Graphpad	Prism v8	https://www.graphpad.com/scientific-software/prism/
ggplot2 3.3.5	(Wickham et al., 2020)	https://cran.r-project.org/web/packages/ggplot2/index.html
ROntoTools 2.16.0	(Voichita et al., 2020)	https://bioconductor.org/packages/3.11/bioc/html/ROntoTools.html
ROCR 1.0-11	(Sing et al., 2005)	https://cran.r-project.org/web/packages/ROCR/index.html
GeoDE 1.0	(Clark et al., 2014)	https://cran.rstudio.com/web/packages/GeoDE/index.html
Limma 3.44.3	(Ritchie et al., 2015)	https://bioconductor.org/packages/3.11/bioc/html/limma.html
RobustRankAggreg 1.1	(Kolde et al., 2012)	https://cran.r-project.org/web/packages/RobustRankAggreg/index.html
enrichR 3.0	(Kuleshov et al., 2016)	https://cran.r-project.org/web/packages/enrichR/index.html

**Other**		

Nuclear Magnetic Resonance (NMR) Bruker minispec series mq10 machine	Bruker BioSpin	

### Resource availability

#### Lead contact

Further information and requests for resources should be directed to and will be fulfilled by the lead contact, Xia Yang (xyang123@ucla.edu).

#### Materials availability

This study did not generate new reagents.

### Experimental model and subject details

#### Animals

Since drug repositioning was done using steatosis gene signatures, we validated the predicted drugs using a diet-induced steatosis mouse model which has been previously (Hui et al., 2015; Chella Krishnan et al., 2018, 2021; Norheim et al., 2021) used to study NAFLD. Briefly, seven-week old C57BL/6J male mice were purchased from the Jackson Laboratory (Bar Harbor, ME). Mice were maintained on a 12-hour light/dark cycle environment at UCLA and were given *ad libitum* access to food and water. After a one week acclimation period mice were randomly assigned to four experimental groups (n=7-9/group) on different diets/treatments: regular chow diet (Control) (Lab Rodent Diet 5053, St. Louis, MO), high-fat high-sucrose (HFHS) diet (Research Diets-D12266B, New Brunswick, NJ) to induce hepatic steatosis, a key NAFLD phenotype, HFHS diet with Fluvastatin treatment (NAFLD + Flu), and HFHS diet with aspirin treatment (NAFLD + Asp). All animal experiments were done under the protocol approved by the UCLA institutional animal care and use committee (IACUC).

### Method details

#### Curation of tissue- and species-specific drug transcriptomic datasets

A total of 941 drugs, including 766 FDA approved drugs from KEGG, FDA, European Medical Agency, and Japanese Pharmaceuticals and Medical Devices Agency, and 175 chemicals from TG-GATEs and DrugMatrix were queried against GEO, ArrayExpress, TG-GATEs, and DrugMatrix to identify datasets. Duplicated datasets between data repositories were removed. We developed a semi-automated pipeline combining automated search with manual checking to identify relevant datasets for drug treatment. The automated process first extracts datasets containing drug generic names or abbreviations and then inspects the potential datasets for availability of both drug treatment and control labels in the constituent samples. Labels identified by the automated process were also manually checked to validate the labels and remove potential false detections. Only datasets with n>=3/group in both drug treatment and control groups were included in our downstream analyses. Although a larger sample size is desired, the majority (77.7%) of drug transcriptome datasets for the dose/time segregated signature database have n=3/group, 21.9% datasets have n=2/group, and <1% datasets have n>3/group (Figure S4A). It should be noted that this sample size is used in all major drug/chemical signature databases, including CMap, L1000, TG-GATEs and DrugMatrix, in order to cover different chemicals and time and dose regimens. GEO/ArrayExpress datasets showed larger sample size variation compared to dedicated toxicogenomics databases (Figure S4B). Currently our gene signatures were obtained from microarray datasets since RNA-seq datasets were not standardized in the GEO/ArrayExpress platform and different normalization methods will require a different downstream processing pipeline. The 1460 microarray datasets for 342 drugs from GEO/ArrayExpress were from Affymetrix (55%), Illumina (25%) and Agilent (20%) platforms; the 5370 DrugMatrix datasets for 655 drugs and chemicals contained Affymetrix and Codelink microarrays; the 6700 datasets for 169 drugs and chemicals from TG-GATEs mainly used Affymetrix microarrays. Affymetrix and Illumina microarrays provided similar transcriptome coverages while Codelink platform is an older design which only covered around 6000 genes. Agilent microarrays are two-color compared to the other three platforms which used single-color arrays.

#### Obtaining drug treatment signatures stratified by species and tissues

Species and tissue labels were retrieved based on the metadata of each dataset. Tissue names were standardized based on the BRENDA Tissue Ontology (Gremse et al., 2011). We implemented a search procedure to climb the ontology tree structure to determine the suitable tissue annotations. This was done by first building a priority list of widely used tissues/organs in toxicological research using the BRENDA Tissue Ontology tree system. Tissue/organ priority order was set to “kidney”, “liver”, “pancreas”, “breast”, “ovary”, “adipose tissue”, “cardiovascular system”, “nervous system”, “respiratory system”, “urogenital system”, “immune system”, “hematopoietic system”, “skeletal system”, “integument” (endothelial and skin tissue), “connective tissue”, “muscular system”, “gland”, “gastrointestinal system”, and “viscus” (other non-classified tissue). Tissue terms relevant to each of these tissues or organs were curated from the ontology tree into a tissue/organ ontology table. Next, we looked up terms from our tissue/organ ontology table in the Cell/Organ/Tissue column of the metadata in each transcriptomic dataset. If a tissue/organ term was not found, we searched the title and summary columns of the metadata as well to retrieve additional information. When the search returned multiple tissue terms (for example, breast cancer cell line may be categorized as both epithelial and breast organ), we used the term with the highest priority as described above. We prioritized the tissue terms based on the relevance to toxicology to make tissue assignments unique for each dataset to reduce ambiguity. Manual checking was conducted to confirm the tissue annotation for each dataset.

For each gene expression dataset from GEO and ArrayExpress, normalized data were retrieved, and quantile distribution of the values was assessed. When a dataset was not normally distributed, log2-transformation using GEO2R (Barrett et al., 2013) was applied. For gene expression datasets from Codelink microarrays (DrugMatrix), quantile normalization was conducted. For Affymetrix microarrays (DrugMatrix and TG-GATEs), GCRMA (Wu et al., 2004) normalization was conducted. To identify differentially expressed genes (DEGs) representing drug signatures, two different strategies were used. First, the widely used DEG analysis method LIMMA (Ritchie et al., 2015) was applied to obtain dose and time segregated signatures under false discovery rate (FDR) < 0.05. To overcome the low sample size issue and obtain “consensus” drug signatures for a drug/chemical, LIMMA was also applied to datasets where multiple doses and treatment durations were tested, and treatment effects were derived by combining dose/time experiments for the same drug/chemical in each study. Second, we leveraged different studies for the same drugs or chemicals in the same tissue and species to derive meta-analysis signatures. To address heterogeneity in study design, platforms, sample size, and normalization methods across different data repositories, we applied the characteristic direction method from the GeoDE package to derive consistent DEGs for each drug across different data sources. GeoDE was designed to accommodate heterogenous datasets that have differing parameters and outputs between treatment and control groups. It uses a “characteristic direction” measure to identify biologically relevant genes and pathways. The normalized characteristic directions for all genes were then transformed into a non-parametric rank representation. Subsequently, gene ranks of a particular drug from the same tissue/organ system and the same organism were aggregated across datasets using the Robust Rank Aggregation method (Kolde et al., 2012), a statistically controlled process to identify drug DEGs within each tissue for each species. Robust Rank Aggregation provides a non-parametric meta-analysis across different ranked lists to obtain commonly shared genes across datasets, which avoids statistical issues associated with heterogeneous datasets. It computes a null distribution based on randomized gene ranks and then compares the null distribution with the empirical gene ranks to obtain a p-value for each gene. The robust rank aggregation process was done for the upregulated and downregulated genes separately to obtain DEGs for both directions under Bonferroni-adjusted p-value < 0.01, a cutoff implemented in the Robust Rank Aggregation algorithm. To obtain species-level signatures for each drug, we further aggregated DEGs across different organs tested for each drug within each species.

Pathway analysis of individual drug signatures was conducted using Enrichr (Kuleshov et al., 2016) by intersecting each signature with pathways or gene sets from KEGG (Kanehisa et al., 2017) and gene ontology biological process (GOBP) terms (The Gene Ontology Consortium, 2017). Gene signatures were defined as FDR < 0.05 for dose/time segregated signatures and Bonferroni-adjusted p-value < 0.01 for meta-analysis signatures. In addition, pathway enrichment analysis based on network topology analysis (Draghici et al., 2007) was conducted using Bioconductor package ROntoTools (Voichita et al., 2020). Pathways at FDR < 0.05 were considered significant in both methods.

We curated 14,366 drug signatures segregated by treatment dosage and duration, tissue, and species, covering 719 drugs and chemicals, among which 554 are FDA approved. In addition, our meta signatures is a consensus of 4,344 signatures covering 551 drugs across treatment regimens. In total, the entire database is based on 13,530 rat, human, and mouse transcriptomic datasets across >20 tissue or organ systems across 941 drugs and chemicals from GEO, ArrayExpress, DrugMatrix, and TG-GATEs to derive drug signatures. The toxicogenomics databases TG-GATEs and DrugMatrix mainly contain liver and kidney datasets from rats, while public data repositories GEO and ArrayExpress contain datasets with broader tissue and species coverage (Figure 1B). Overall, the rat datasets are mainly from liver and kidney whereas human and mouse datasets also contained signatures from other tissues and organs such as breast and the nervous system (Figure 1C). There is also a species bias between the data repositories; GEO covered more mouse and human datasets, TG-GATEs mainly has human and rat datasets, and DrugMatrix curated more rat datasets (Figure 1D).

#### Curation of gene networks

We used tissue-specific networks, for example Bayesian gene regulatory networks (BNs) of mouse liver constructed using a previously established method (Zhu et al., 2007, 2008) based on transcriptomic and genetic data from different mouse liver transcriptomic datasets (Yang et al., 2006; Wang et al., 2007; Derry et al., 2010; Zhong et al., 2010; Tu et al., 2012). For each dataset, 1,000 BNs with different random seeds were reconstructed using Monte Carlo Markov Chain simulation and the model with the best fit for each network was determined. In the resulting set of 1,000 networks, edges appearing in over 30% of the networks were included in a consensus network. This practice has been found to produce experimentally supported regulatory relations between genes (Zhu et al., 2007, 2008). The union of nodes and edges from BNs of multiple mouse or human studies were used as tissue-specific networks.

#### Curation of drug signatures from CMap, LINC1000, and CREEDS for comparison with PharmOmics

To compare PharmOmics with other established drug signature platforms for drug repurposing, we downloaded signatures from L1000FWD (Clark et al., 2014) (http://amp.pharm.mssm.edu/l1000fwd/download_page) which were well annotated to match drug signatures for comparison. For CREEDS (Wang et al., 2016a) (http://amp.pharm.mssm.edu/CREEDS/) repositioning, the web-based Enrichr (Kuleshov et al., 2016) tool was used to query disease signatures to their DrugMatrix library, and outputs based on “combined score” implemented by Enrichr were used. Finally, CMap repositioning test were completed through query from the website directly (https://clue.io/) and rank based CMap scoring was used. For CMap and L1000 results which are based on *in vitro* cell lines, results from all cell lines were summarized to represent common usage of *in vitro* studies. For CREEDS results where *in vivo* studies were available, only the corresponding tissues were included for comparability with PharmOmics. We compared PharmOmics with the CREEDS, CMap and L1000 at the regimens that showed the best performance in drug repurposing analysis in each platform.

#### Curation of disease gene signatures for drug repositioning

To test the potential of PharmOmics drug signatures for drug repositioning, we curated disease gene signatures for hyperlipidemia and NAFLD. Hyperlipidemia was chosen as a test disease because numerous positive control drugs are available to assess the performance of PharmOmics in retrieving known drugs compared to other existing drug repositioning tools. NAFLD was chosen as another test case since no effective drugs are currently available for this condition and our predictions may help guide future drug development.

The hyperlipidemia signatures were derived from two resources: i) genes and pathways identified by the Mergeomics pipeline (Shu et al., 2016) based on low-density lipoprotein cholesterol (LDL) genome-wide association study (GWAS) summary statistics data (Willer et al., 2013), and ii) genes based on mechanistic and therapeutic evidence collected by the Comparative Toxicogenomics Database (CTD) (Davis et al., 2017) under Mesh ID D006949. These two different resources represent disease gene signatures derived from either GWAS inference or a literature-based system. NAFLD gene signatures were retrieved from i) studies of NAFLD mouse model (Chella Krishnan et al., 2018) from a large systems genetics cohort comprised of hundreds of mice from ∼100 genetically diverse strains, and ii) CTD gene signature under Mesh ID D065626.

As additional test cases, we also retrieved gene signatures for chemical induced liver injury under CTD Mesh ID D056486, for hepatitis under CTD Mesh ID D006527, for hyperuricemia under CTD Mesh ID D033461, and for type 2 diabetes under CTD Mesh ID D003924.

#### Measurement of similarity between signatures of drugs, ADRs, and diseases

We used two different methods to determine similarities between two signatures (e.g., a drug signature vs. a disease or ADR signature, or a drug signature vs. signature of another drug). The first method is based on signature overlaps and uses a signed Jaccard score based on upregulated genes from the first signature set (*a1*), upregulated genes from the second signature set (*b1*), downregulated genes from the first signature set (*a2*) and downregulated genes from the second signature set (*b2*). The Jaccard score was defined in the following formula:

$$J\left(A,B\right)=\frac{\left|A\cap B\right|}{\left|A\cup B\right|}$$

signedJaccardscore=J(a1,b1)+J(a2,b2)−J(a1,b2)−J(a2,b1)

If there is no direction from the disease signature (*a*), Jaccard score was determined based on a simple overlap between disease signature (*a*) and drug signatures (*b*) without considering direction (*b1*, *b2*).

The second method to determine similarities between two signatures is based on a distance measure derived from the mean of shortest path lengths between network key drivers of a drug gene signature (A) and a disease signature (B) in a given Bayesian gene regulatory network (BN) based on a key driver analysis (see below for details). This distance measure is adapted from a previous study using protein interaction networks (Cheng et al., 2018).

$$\text{distance}\left(\text{B},\;\text{A}\right)\;=\;\frac{1}{\left\|A\right\|}\sum_{a\in A}\text{min}\;\text{b}\;\in \;\text{B}\;\text{ distance}\;\left(\text{b},\;\text{a}\right)$$

To reduce variation, only signatures with more than 10 genes were included in the analysis. To obtain a null distribution for shortest path lengths, we permuted genes with the same degree as the drug/disease/ADR genes in each network 1,000 times and calculated a z-score based on the mean and standard error of the null distribution.

Comparison of gene signatures from different species used gene symbol conversion based on ortholog information from HGNC consortium (Tweedie et al., 2021). The ROCR package (Sing et al., 2005) was used to assessed the performance of the gene overlap based or network based methods in drug repositioning or ADR prediction.

#### Comparison of PharmOmics with existing drug signature platforms

To assess the degree of agreement in drug signatures between the PharmOmics database and existing platforms, we compared PharmOmics with the CREEDS (Wang et al., 2016a) and L1000FWD (Wang et al., 2018) databases, for which drug signatures are accessible. As shown in Figure S3, both the PharmOmics dose/time-segregated signatures and the meta signatures showed better concordance with the two existing platforms than the agreement between CREEDS and L1000FWD, as reflected by higher overlap fold enrichment score and lower statistical p values. These platforms have differences in the datasets and analytical strategies and therefore are complementary. Due to the lack of full access to CMap signatures, we were not able to systematically compare PharmOmics against CMap.

#### PharmOmics web server implementation

To allow easy data access and use of PharmOmics, we have created a freely accessible web tool deployed on the same Apache server used to host Mergeomics (Shu et al., 2016), a computational pipeline for integrative analysis of multi-omics datasets to derive disease-associated pathways, networks, and network regulators (http://mergeomics.research.idre.ucla.edu).

The PharmOmics web server features three functions (Figure 2A). First, it allows queries for species- and tissue-stratified drug signatures and pathways for both the dose/time-segregated and meta signatures. Details of statistical methods (e.g., LIMMA vs. characteristic direction), signature type (dose/time-segregated vs. meta), and datasets used are annotated. The drug query also includes a function for DEG and pathway signature comparisons between user-selected species and tissues which can be visualized and downloaded. Second, it features a network drug repositioning tool that is based on the connectivity of drug signatures in PharmOmics to user input genes such as a disease signature. This tool requires a list of genes and a gene network that can be chosen from our preloaded gene regulatory networks if relevant or a custom upload (see Applications below for details in implementation). In order to keep reasonable computation time and memory requirement of network repositioning on dose/time segregated signatures, we implemented on the web server the option to run repositioning with a maximum of 500 genes for each drug signature, which were defined by their FDR value regardless of directionality. In the output, Z-score and p-value results of network repositioning are displayed and available for download. In addition, we list the overlapping genes between drug signatures in the given network and the input genes, the drug genes with direct connections to input genes through one-edge extension, and input genes with one-edge connections to drug genes in the downloadable results file. The output page also provides network visualization which details the genes affected by a drug and their overlap with and direct connections to user input genes using Cytoscape.js. The network nodes and edges files are also available for download and can be used on Cytoscape Desktop. An example of the web interface of the input submission form and results display of the network repositioning tool using a sample liver network and a sample hyperlipidemia gene set is shown in Figures 2B and 2C. Lastly, the web server offers a gene overlap-based drug repositioning tool that assesses direct overlap between drug gene signatures and user input genes. Gene overlap-based drug repositioning requires a single list of genes or separate lists of upregulated and downregulated genes and outputs the Jaccard score, odds ratio, Fisher’s exact test p-value, within-species rank, and gene overlaps for drugs showing matching genes with the input genes. This gene overlap-based approach is similar to what was implemented in other drug repositioning tools, but the network-based repositioning approach is unique to PharmOmics.

#### Experimental methods for NAFLD drug validation

Eight week old mice underwent dietary treatment with fluvastatin and aspirin purchased from Cayman Chemicals (Ann Arbor, MI). The target intake concentrations of fluvastatin and aspirin were 15 mg/kg and 80 mg/kg, respectively, which were chosen based on doses used in previous studies that did not show toxicity (Park et al., 2016; Zhu et al., 2017). These experimental diets were then administered for 10 weeks. The average fluvastatin intake was 14.98 mg/kg/day, and the average aspirin intake was 79.67 mg/kg/day. During drug treatment, metabolic phenotypes such as body weight, body fat and lean mass composition were monitored weekly. Fat and lean mass were measured with Nuclear Magnetic Resonance (NMR) Bruker minispec series mq10 machine (Bruker BioSpin, Freemont, CA). At the end of treatment, mice were sacrificed after a 4 hour fasting period and livers from all groups were weighed, flash frozen, and stored at −80°C until lipid analysis. For metabolic phenotypes measured at multiple time points (body weight gain and adiposity), differences between groups were analyzed using a 2-way ANOVA followed by Sidak’s multiple comparisons test.

#### Hepatic lipid quantification

Hepatic lipids were extracted using the Folch method (Folch etal., 1957). Briefly, frozen liver tissues were homogenized in methanol, and then chloroform was added to each sample to obtain a 2:1 mixture of chloroform and methanol. Samples were then incubated overnight at 4C. Following incubation, samples were filtered and magnesium chloride was added to the filtrate and centrifuged. The resulting aqueous phase and soluble proteins were aspirated, and the remaining organic phase was evaporated using nitrogen gas. The dried lipids were dissolved in a Triton X-100 solution. The samples were stored in −80°C prior to analysis. The lipid extracts were analyzed by the UCLA GTM Mouse Transfer Core for triglyceride (TG), total cholesterol (TC), unesterified cholesterol (UC), and phospholipids (PL) levels by colorimetric assays (Warnick, 1986; Hedrick et al., 1993). Depending on data normality, the groups were analyzed using either a two-sided t-test or Mann-Whitney test.

### Quantification and statistical analysis

Data representation, dispersion and precision measures can be viewed in the figure legends. For *in vivo* experimental data comparisons using two-way ANOVA (with Sidak post-hoc analysis), t-test and Mann-Whitney test, Prism v8 was used for analysis. Significance level p < 0.05 is noted using an asterisk ∗. For repositioning score two group comparison was performed by Wilcoxon signed rank test in R 4.0.2. Significance levels p < 0.05, p < 0.01 and p < 0.001 are noted using asterisks ∗, ∗∗, and ∗∗∗, respectively. Multiple group comparison was performed by Kruskal-Wallis test followed by post-hoc pairwise Wilcoxon signed rank test in R 4.0.2. Significance levels p < 0.05, p < 0.01 and p < 0.001 are noted using asterisks ∗, ∗∗, and ∗∗∗, respectively). Figures were generated by Prism v8 for *in vivo* experimental data, R default plot for ROC curves and histograms, and R ggplot2 (Wickham et al., 2020) for boxplots. Sample sizes can be viewed in the figure legends. The statistics used in the bioinformatics analysis was described in the individual method sections above.

## Data Availability

• All data, including indexed dataset catalog, pre-computed drug signatures and pre-computed pathway enrichments for individual drugs are deposited to and accessible through the PharmOmics web server (http://mergeomics.research.idre.ucla.edu/runpharmomics.php). We also implemented functions for same-tissue between-species comparison and same-species between-tissue comparison and comparison result download. In addition, network-based drug repositioning analysis and gene overlap-based drug repositioning analysis using all drug signatures are available at http://mergeomics.research.idre.ucla.edu/runpharmomics.php. • Code for PharmOmics repositioning is available at https://github.com/XiaYangLabOrg/pharmomics. • Any additional information required to reanalyze the data reported in this paper is available from the lead contact upon request.

## Supplemental information

Document S1. Figures S1–S4 and Tables S1–S3

Table S4. Network repositioning result for non-alcoholic fatty liver disease based on genetic pathways obtained from studies of female and male mice, related to Figure 4

Table S5. Submodule repositioning result based on signatures from CTD chemical induced liver injury, related to Figure 5

Table S6. Cross-tissue comparison of Atorvastatin Pathways, related to Figure 6

Table S7. Cross-species comparison of Rosuvastatin Pathways, related to Figure 6
