# Supplementary material for: PharmOmics: A species- and tissue-specific drug signature database and gene-network-based drug repositioning tool
Source: iScience. 2022 Mar 10;25(4):104052. doi: 10.1016/j.isci.2022.104052 (PMC8957031; doi:10.1016/j.isci.2022.104052)
Supplement: Document S1. Figures S1–S4 and Tables S1–S3 [file mmc1.pdf]

iScience, Volume *25*

Supplemental information

PharmOmics: A species- and tissue-specific drug signature database and gene-network-based drug repositioning tool

Yen-Wei Chen, Graciel Diamante, Jessica Ding, Thien Xuan Nghiem, Jessica Yang, Sung-Min Ha, Peter Cohn, Douglas Arneson, Montgomery Blencowe, Jennifer Garcia, Nima Zaghari, Paul Patel, and Xia Yang

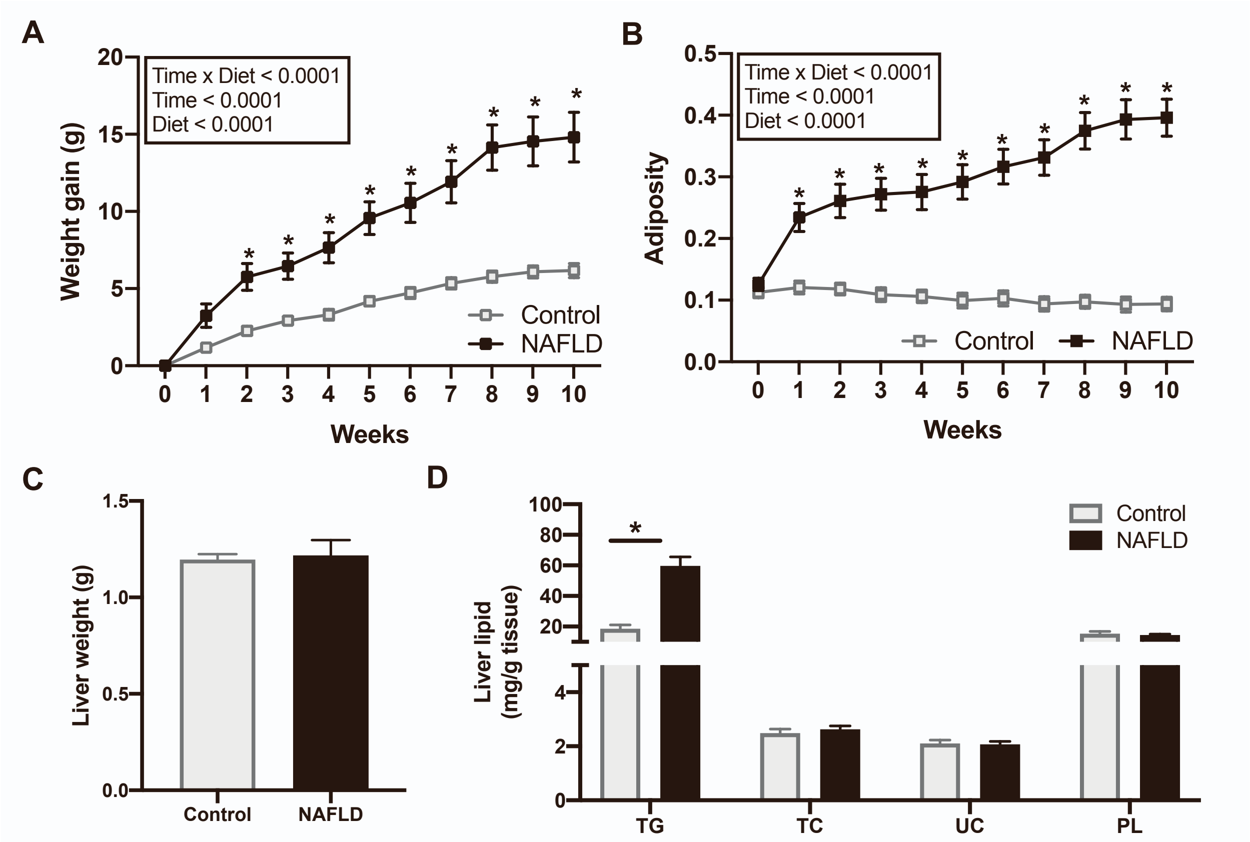

Figure S1. Effects of high fat high sucrose diet on body composition and liver lipids in C57BL/6J mice, Related to Figure 4. (A) Time course of body weight gain of mice on control and high fat high sucrose (HFHS) diet for 10 weeks. (B) Time course of adiposity of mice on control and HFHS diet for 10 weeks. (A and B) Data are represented as mean +/- SEM and was analyzed by two-way ANOVA followed by Sidak post-hoc analysis to examine treatment effects at individual time points. (C) Bar plot of liver weight in mice on a control and HFHS diet. (D) Bar plot of hepatic lipid levels in mice on a control and HFHS diet (D). Triglyceride (TG), Total Cholesterol (TC), Unesterified Cholesterol (UC), Phospholipid (PL). (C and D) Data are represented as mean +/- SEM and was analyzed using either the two-sided t-test or Mann-Whitney test. P value <0.05 was considered significant and is denoted by an asterisk (*). Sample size n = 8-9/group. Control diet (Control); HFHS diet (NAFLD).

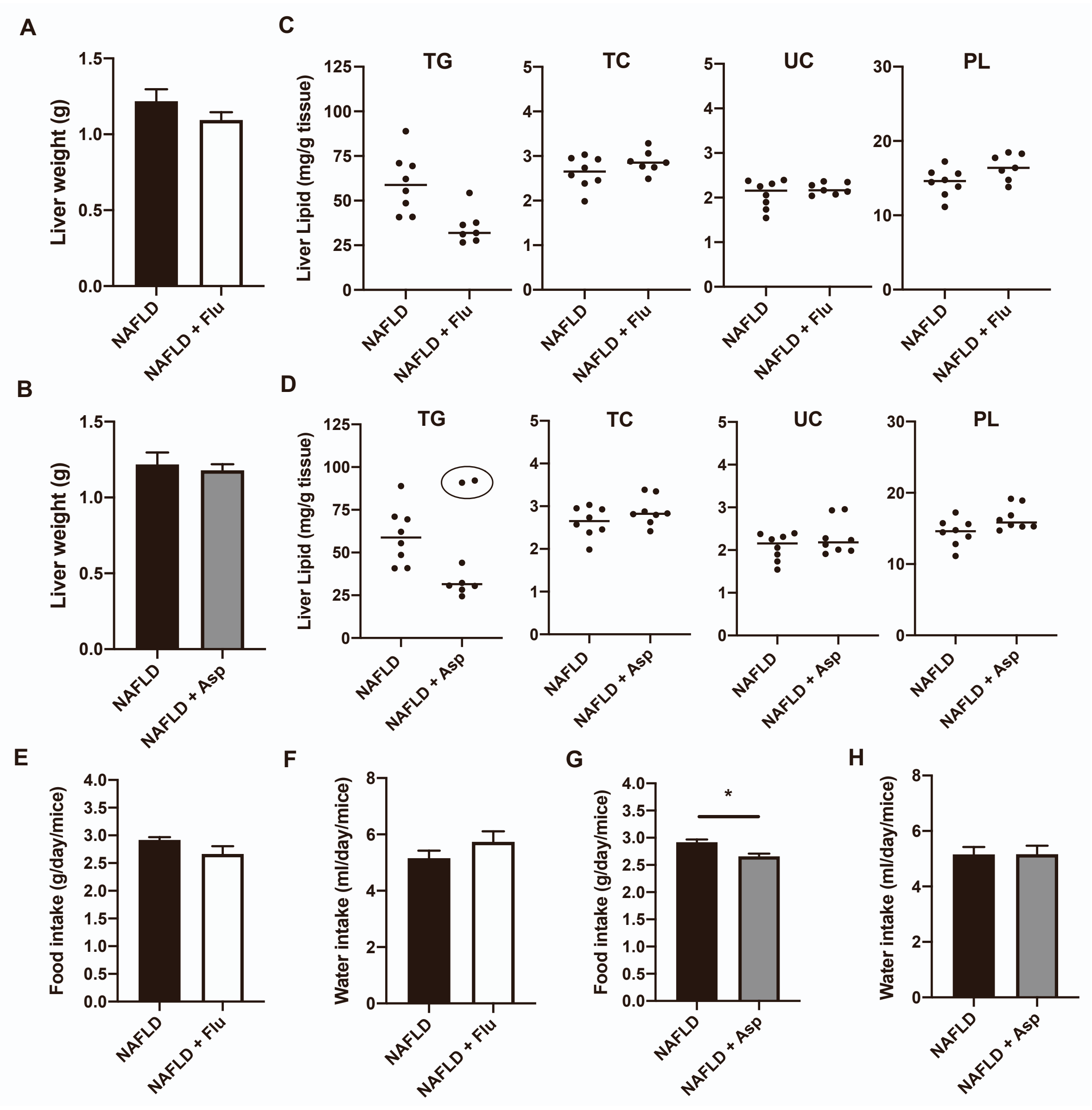

Figure S2. Liver weight, food intake, and water intake in C57BL/6J mice on a HFHS diet with or without fluvastatin or aspirin, Related to Figure 4. (A and B) Bar plot of liver tissue weight in mice on a HFHS diet with or without fluvastatin (A) or aspirin (B). (C and D) Dotplots of lipid levels in the liver of mice on fluvastatin (C) or aspirin (D) treatment for 10 weeks. Triglyceride (TG), Total Cholesterol (TC), Unesterified Cholesterol (UC), Phospholipid (PL). Circle indicates the identified outliers using the ROUT method on Graphpad (Prism v8), which were removed from the subsequent analysis. (E and F) Bar plot of food (E) and water (F) intake in mice on a HFHS diet with or without fluvastatin. (G and H) Bar plot of food (G) and water (H) intake in mice on a HFHS diet with or without aspirin. (A, B and E-H) Data are represented as mean +/- SEM. All data was analyzed using a two-sided t-test. P value <0.05 was considered significant and is denoted by an asterisk (*). Sample size n = 7-8/group. HFHS group (NAFLD); HFHS with Fluvastatin (NAFLD + Flu); HFHS with Aspirin (NAFLD + Asp).

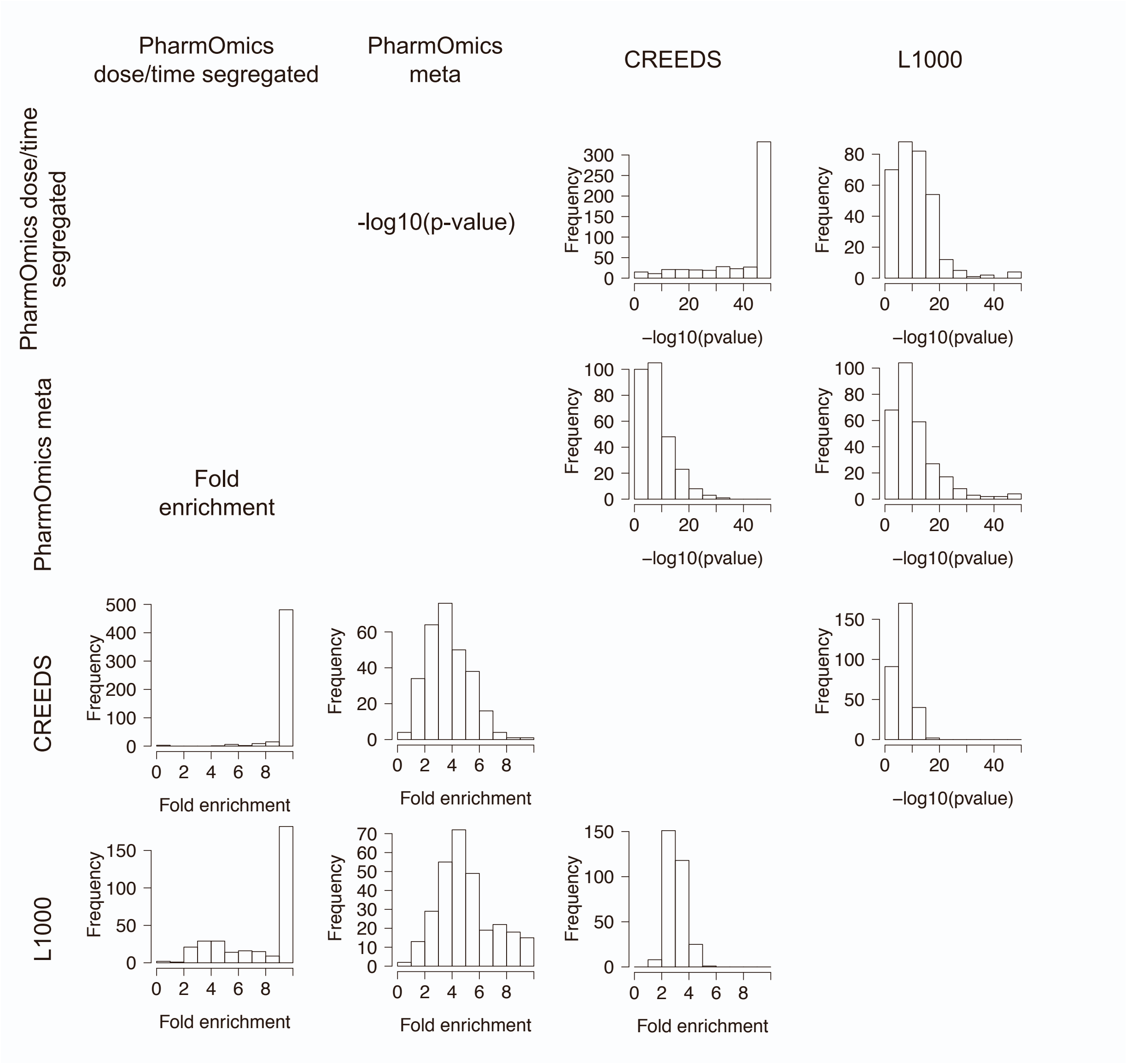

Figure S3. Comparison of drug signatures between PharmOmics and existing drug signature databases CREEDS and L1000, Related STAR Methods. For each drug in PharOmics database shared among other databases, only the overlap scores for the best matched signatures between two databases are used. Lower left triangular matrix represents the histogram of the gene overlap fold enrichment scores calculated using hypergeometric test, and the upper right triangular matrix represents the histogram of the -log10(pvalue) from Fisher's exact test.

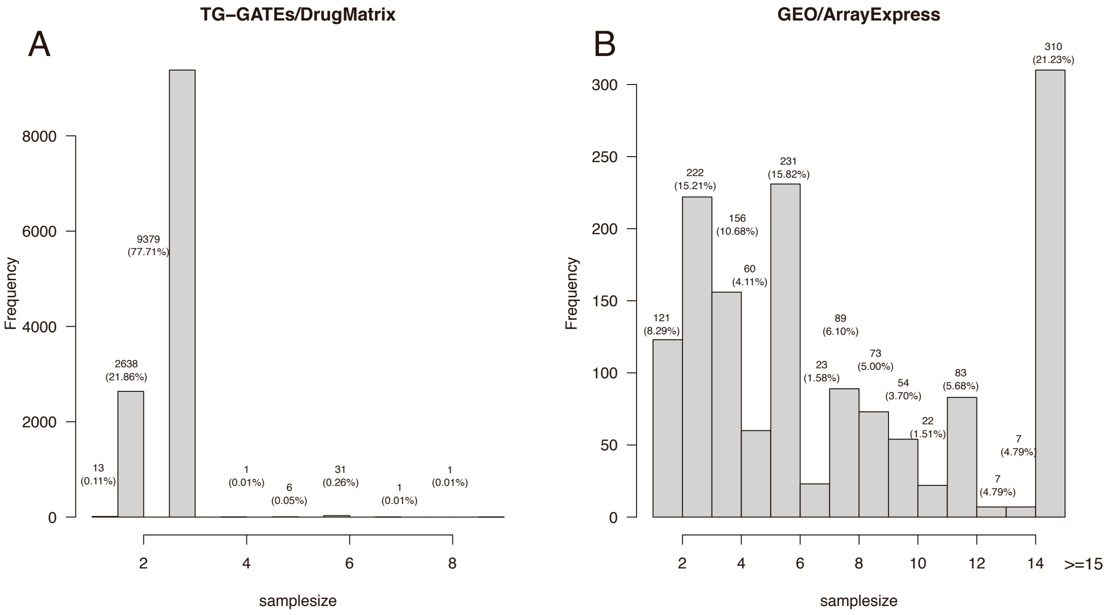

Figure S4. Histogram of sample size distribution among different PharmOmics signature databases, Related to STAR Methods. (A) Sample size distribution of datasets from TG-GATEs and DrugMatrix. (B) sample size distribution of datasets from GEO/Arrayexpress database. Datasets with sample size <3/group were excluded from downstream analysis.

Table S1. Prediction percentile of steroid and non-steroid anti-inflammatory drugs based on hepatitis signatures from CTD database across different platforms tested, Related to Figure 3. HEPG2 results from both L1000 and CMap were retrieved for tissue specificity comparison.

Drugname	PharmOmicsdose/time seg Network	PharmOmicsdose/time seg Jaccard	CREEDS	CMap_HEPG2	Cmap	L1000_HEPG2	L1000
Aspirin	0.870	0.874	0.856	NA	0.102	NA	NA
Betamethasone	0.959	0.623	0.372	0.945	0.697	0.941	0.877
Bromfenac	0.618	0.774	0.705	NA	0.777	NA	NA
Cortisone	0.515	0.146	0.093	0.399	0.393	NA	0.550
Dexamethasone	0.417	0.911	0.968	0.733	0.678	0.552	0.956
Diclofenac	0.593	0.821	0.521	0.204	0.503	NA	0.645
Fluocinolone acetonide	0.428	NA	NA	NA	NA	NA	NA
Hydrocortisone	0.978	0.804	0.422	0.676	0.681	NA	0.810
Ibuprofen	1.000	0.898	0.972	NA	0.390	NA	0.436
Indomethacin	0.913	0.753	0.989	NA	NA	NA	NA
Ketorolac	0.780	0.889	0.993	0.438	0.791	NA	0.309
Mefenamic acid	0.924	0.694	NA	NA	NA	NA	NA
Naproxen	0.569	0.472	0.594	0.753	0.937	NA	0.340
Phenylbutazone	0.976	0.967	NA	0.066	0.592	NA	0.185
Prednisolone	0.911	0.840	0.258	0.714	0.759	0.219	0.858
Sulindac	0.932	0.724	0.872	NA	0.286	NA	0.386
Acemetacin	NA	NA	0.270	NA	NA	NA	NA
Alclometasone	NA	NA	NA	0.219	0.188	0.706	0.546
Amcinonide	NA	NA	NA	0.241	0.560	0.284	0.717
Beclometasone	NA	NA	NA	0.498	0.018	0.088	0.734
Benzydamine	NA	NA	NA	0.120	0.484	NA	0.453
Budesonide	NA	NA	NA	0.322	0.636	0.552	0.627

Clobetasol	NA	NA	NA	0.928	0.675	0.729	0.844
Desoximetasone	NA	NA	NA	0.289	0.319	0.830	0.527
Dexketoprofen	NA	NA	NA	0.256	0.327	NA	0.231
Diflorasone	NA	NA	NA	0.963	0.991	NA	0.673
Diflunisal	NA	NA	NA	0.781	0.350	NA	0.720
Fenbufen	NA	NA	NA	0.889	0.353	NA	0.015
Flunisolide	NA	NA	NA	0.247	0.929	NA	0.978
Fluticasone	NA	NA	NA	0.729	0.787	0.529	0.899
Halcinonide	NA	NA	NA	0.216	0.363	NA	0.335
Ketoprofen	NA	NA	NA	0.076	0.808	NA	0.015
Medrysone	NA	NA	NA	0.663	0.737	NA	0.923
Methylprednisolone	NA	NA	NA	0.381	0.042	0.088	0.164
Mometasone	NA	NA	NA	0.987	0.906	NA	0.265
Oxaprozin	NA	NA	NA	0.633	0.418	NA	0.179
Piroxicam	NA	NA	NA	0.034	0.898	NA	0.851
Prednicarbate	NA	NA	NA	0.238	0.556	0.088	0.920
Prednisone	NA	NA	NA	0.393	0.952	NA	0.622
Rimexolone	NA	NA	NA	0.214	0.441	NA	0.542
Tenoxicam	NA	NA	NA	0.694	0.340	NA	0.238
Tolmetin	NA	NA	NA	0.870	0.273	NA	0.138
Triamcinolone	NA	NA	NA	0.817	0.701	NA	0.807
Ampiroxicam	NA	NA	NA	NA	0.203	NA	0.043
Fluocinonide	NA	NA	NA	NA	0.217	NA	0.609
Fluorometholone	NA	NA	NA	NA	0.574	NA	0.687
Halometasone	NA	NA	NA	NA	0.756	NA	0.553
Loteprednol	NA	NA	NA	NA	0.658	NA	0.665
Loxoprofen	NA	NA	NA	NA	0.911	NA	0.392
Mofezolac	NA	NA	NA	NA	0.854	NA	0.330
Nabumetone	NA	NA	NA	NA	0.685	NA	0.309

Clocortolone	NA	NA	NA	NA	NA	NA	0.976
Flurbiprofen	NA	NA	NA	NA	NA	NA	0.818
**Median**	**0.890**	**0.804**	**0.649**	**0.438**	**0.592**	**0.541**	**0.553**
**Mean**	**0.774**	**0.746**	**0.635**	**0.504**	**0.564**	**0.467**	**0.547**

Table S2. Prediction percentile of FDA approved anti-diabetic drug based on type2 diabetes signatures from CTD database across different platforms tested, Related to Figure 3. HEPG2 results from both L1000 and CMap were retrieved for tissue specificity comparison.

**Drugname**	**PharmOmics dose/time seg** **Network**	**PharmOmicsdose/time seg** **Jaccard**	**CREEDS**	**CMap_HEPG2**	**Cmap**	**L1000_HEPG2**	**L1000**
**Sulfonylurea drugs**							
Chlorpropamide	0.547	0.279	NA	NA	NA	NA	NA
Glimepiride	0.241	0.45	0.456	0.835	0.343	NA	0.717
Glipizide	0.26	0.301	0.523	0.498	0.845	NA	0.005
Nateglinide	0.179	0.48	0.231	0.289	0.59	NA	0.56
Tolazamide	0.046	0.397	0.267	0.465	0.12	NA	0.77
Tolbutamide	0.699	0.136	NA	0.832	0.29	NA	0.361
Gliquidone	NA	NA	NA	0.94	0.678	NA	0.27
Repaglinide	NA	NA	NA	NA	0.779	NA	0.376
**Median**	**0.251**	**0.349**	**0.361**	**0.665**	**0.59**	**NA**	**0.376**
**Mean**	**0.329**	**0.34**	**0.369**	**0.643**	**0.521**	**NA**	**0.437**
**PPAR gamma agonist**							
Pioglitazone	0.688	0.298	0.854	0.906	0.656	0.35	0.826
Rosiglitazone	0.81	0.734	0.765	0.955	0.97	NA	0.68
Troglitazone	0.873	0.419	0.655	0.684	0.256	NA	0.976
Ciglitazone	NA	NA	NA	0.499	0.925	NA	0.023
**Median**	**0.81**	**0.419**	**0.765**	**0.795**	**0.791**	**0.35**	**0.753**
**Mean**	**0.79**	**0.484**	**0.758**	**0.761**	**0.702**	**0.35**	**0.626**

Table S3. Prediction percentile of FDA approved gout treatment drug based on hyperuricemic signatures from CTD database across different platforms tested, Related to Figure 3. HEPG2 results from both L1000 and CMap were retrieved for tissue specificity comparison.

**Drugname**	**PharmOmics dose/time seg<br><br>Network**	**PharmOmics dose/time seg<br><br>Jaccard**	**CREEDS**	**CMap_ HEPG2**	**Cmap**	**L1000_ HEPG2**	**L1000**
Allopurinol	0.846	0.317	NA	NA	NA	NA	NA
Benzbromarone	0.946	0.451	NA	0.313	0.475	0.408	0.895
Colchicine	0.889	0.271	NA	NA	NA	NA	NA
Febuxostat	NA	NA	NA	0.94	0.821	NA	NA
Probenecid	NA	NA	NA	0.043	0.211	NA	0.176
Sulfinpyrazone	NA	NA	NA	NA	0.524	NA	0.176
**Median**	**0.889**	**0.317**	**NA**	**0.313**	**0.499**	**0.408**	**0.176**
**Mean**	**0.893**	**0.346**	**NA**	**0.432**	**0.508**	**0.408**	**0.416**
